# An Immunomodulatory Transcriptional Signature Associated With Persistent *Listeria* Infection in Hepatocytes

**DOI:** 10.3389/fcimb.2021.761945

**Published:** 2021-11-10

**Authors:** Natalie Descoeudres, Luc Jouneau, Céline Henry, Kevin Gorrichon, Aurélie Derré-Bobillot, Pascale Serror, Laura Lee Gillespie, Cristel Archambaud, Alessandro Pagliuso, Hélène Bierne

**Affiliations:** ^1^ Université Paris-Saclay, INRAE, AgroParisTech, Micalis Institute, Jouy-en-Josas, France; ^2^ Université Paris-Saclay, INRAE, Virologie et Immunologie Moléculaires, Jouy-en-Josas, France; ^3^ Université Paris-Saclay, Institut de Biologie Intégrative de la Cellule, CEA, CNRS UMR 9198, Université Paris-Sud, Gif-sur-Yvette, France; ^4^ Terry Fox Cancer Research Laboratories, Division of BioMedical Sciences, Faculty of Medicine, Memorial University of Newfoundland, St. John’s, NL, Canada

**Keywords:** *Listeria monocytogenes*, liver, acute phase response, interferon, persistence, innate immunity, cholesterol, transcriptomics

## Abstract

*Listeria monocytogenes* causes severe foodborne illness in pregnant women and immunocompromised individuals. After the intestinal phase of infection, the liver plays a central role in the clearance of this pathogen through its important functions in immunity. However, recent evidence suggests that during long-term infection of hepatocytes, a subpopulation of *Listeria* may escape eradication by entering a persistence phase in intracellular vacuoles. Here, we examine whether this long-term infection alters hepatocyte defense pathways, which may be instrumental for bacterial persistence. We first optimized cell models of persistent infection in human hepatocyte cell lines HepG2 and Huh7 and primary mouse hepatocytes (PMH). In these cells, *Listeria* efficiently entered the persistence phase after three days of infection, while inducing a potent interferon response, of type I in PMH and type III in HepG2, while Huh7 remained unresponsive. RNA-sequencing analysis identified a common signature of long-term *Listeria* infection characterized by the overexpression of a set of genes involved in antiviral immunity and the under-expression of many acute phase protein (APP) genes, particularly involved in the complement and coagulation systems. Infection also altered the expression of cholesterol metabolism-associated genes in HepG2 and Huh7 cells. The decrease in APP transcripts was correlated with lower protein abundance in the secretome of infected cells, as shown by proteomics, and also occurred in the presence of APP inducers (IL-6 or IL-1β). Collectively, these results reveal that long-term infection with *Listeria* profoundly deregulates the innate immune functions of hepatocytes, which could generate an environment favorable to the establishment of persistent infection.

## Introduction

The liver has essential roles in metabolism and detoxification. It also acts as a barrier to systemic infections, by its major role in the detection, capture and clearance of pathogens present in the blood (Protzer et al., 2012; Kubes and Jenne, 2018). Certain pathogens, including viruses (*e.g.*, Hepatitis A, B, C, D, E viruses), parasites (e.g., *Plasmodium falciparum*, *Toxoplasma gondii*, *Entamoeba histolytica*) and bacteria (e.g., *Listeria monocytogenes*, *Salmonella Typhimurium*, *Francisella tularensis*, *Brucella abortus*, *Streptococcus pneumoniae*) are able to invade hepatocytes, the parenchymal cells of the liver. Among these pathogens, *L. monocytogenes* (hereafter referred to as *Listeria*) is a bacterial food contaminant capable of reaching and multiplying in the liver after crossing the intestinal barrier. In the majority of individuals, invasion of *Listeria* is successfully cleared, but if the infection is not controlled by an adequate immune response, the proliferation of *Listeria* can lead to the release of intracellular bacteria into the circulatory system and invasion of other sites, such as the brain in immunocompromised individuals, and the placenta and the fetus in pregnant women, leading to sepsis, meningo-encephalitis, miscarriages and neonatal infections (Schlech, 2019). These severe clinical manifestations make listeriosis one of the most lethal foodborne infections (European Food Safety, 2021).

Most of our knowledge of the liver phase of listeriosis comes from experimental infections in animal models, mainly mice, resulting in a very well described infection scenario, although some differences may arise from the different routes of inoculation and/or bacterial dose used. After intravenous inoculation, more than 60% of the bacteria are cleared from the bloodstream by the liver within 10 minutes. Bacteria are bound to Kupffer cells (KC, the resident liver macrophages) and subsequently eliminated through a complex interaction between KC and neutrophils that migrate rapidly to the liver in response to infection (Conlan and North, 1991; Gregory et al., 1996; Gregory et al., 2002; Witter et al., 2016). Six hours (h) after infection, about 90% of liver bacteria are associated with hepatocytes, within which bacterial replication takes place for 2-3 days. Thus, *Listeria* loads in the liver increase exponentially before reaching a plateau after 3-4 days of infection, and then decrease with the development of specific immunity (Cousens and Wing, 2000). Bacterial invasion of hepatocytes is proposed to occur *via* two routes: direct internalization or cell-to-cell spread from KC (Dramsi et al., 1995; Gaillard et al., 1996; Appelberg and Leal, 2000). Histology (Conlan and North, 1991) and electron microscopy observations (Gaillard et al., 1996) suggest that bacteria spread in the liver parenchyma using the actin-based motility process described in cellular models *in vitro* (Pilgrim et al., 2003; Kortebi et al., 2017). In line with this, *Listeria actA* mutant strains, which do not produce the actin-polymerization factor *actA*, are three orders of magnitude less virulent compared to wild-type strains in murine models (Domann et al., 1992). The direct passage of bacteria from one hepatocyte to another is proposed to generate infectious foci in which *Listeria* disseminates through the parenchyma, without coming into contact with the humoral effectors of the immune system.

Following the phase of active growth in hepatocytes, the bacterial burden strongly decreases in the liver as a result of potent innate immune responses (Cousens and Wing, 2000). Several cell types contribute to the defense of the infected liver against *Listeria* infection, in particular neutrophils, natural killer (NK) cells, dendritic cells (DC) (Conlan and North, 1991; Gregory et al., 1996; Cousens and Wing, 2000; Arnold-Schrauf et al., 2014; Witter et al., 2016) and KC, whose necroptotic death triggers the recruitment of infiltrating monocytes, which proliferate and differentiate into macrophages at the site of infection (Bleriot et al., 2015). These immune cells work together *via* cell to cell contacts and the secretion of cytokines and chemokines to kill bacteria or inhibit their replication, and to lyse infected hepatocytes. The hepatocytes themselves actively participate in the innate immune response by constitutively producing and secreting a variety of proteins that play an important role in innate immunity, such as complement factors and proteins involved in hemostasis (Zhou et al., 2016). These proteins, whose production increases rapidly and substantially in response to inflammatory stimuli are known as acute phase proteins (APPs) (Gabay and Kushner, 1999; Zhou et al., 2016). Production of pro-inflammatory cytokines by KC, monocytes and neutrophils, in response to *Listeria* infection, stimulates APP production by hepatocytes (Kopf et al., 1994; Kummer et al., 2016). This first wave of non-specific defenses is essential to host survival, with inflammation also contributing to the development of acquired resistance by stimulating the priming and proliferation of cytotoxic T cells, which mediate the protective primary and memory responses against *Listeria* (Pamer, 2004; Qiu et al., 2018).

The propensity of *Listeria* to invade and damage the liver has long been documented in rodents; indeed, among the first names proposed for this bacterium were *Bacillus hepatis* (by Hülphers, in 1911) and *Listerella hepatolytica* (by Pirie, in 1927), based on observations of liver necrosis in rabbits and gerbils. In humans, however, while liver abscesses are described in neonatal listeriosis, clinical symptoms of liver injury due to *Listeria* are rarely reported during invasive listeriosis in adults. Moreover, there is a lack of knowledge on the fate of bacteria in organs, including the liver, during the asymptomatic incubation period, which can be very long in cases associated with pregnancy [up to seventy days (Goulet et al., 2013)]. In addition, asymptomatic carriage of *Listeria* exists in healthy humans (Slutsker and Schucha, 1999), as well as in many farm or wild mammal species (Gray and Killinger, 1966; Yoshida et al., 2000; Leclercq et al., 2014; Hurtado et al., 2017; Parsons et al., 2020), yet our understanding of this asymptomatic carriage, particularly in its hepatic stage, is severely lacking.

Recently, the notion has emerged that, in addition to the well-known phases of active replication and motility in the cytosol of host cells, *Listeria* may enter a quiescent phase in vacuolar compartments, ranging from slow growth to dormancy, which may play an important role in asymptomatic infections (Bierne et al., 2018). These vacuolar niches are particularly formed in liver cells, including hepatic macrophages (Birmingham et al., 2008) and hepatocytes (Kortebi et al., 2017), by distinct mechanisms. In particular, we have shown that in the human hepatocyte cell line HepG2 and in primary human hepatocytes, *Listeria* enters a resting phase in acidic vacuoles, called “*Listeria*-containing vacuoles” (LisCVs) (Kortebi et al., 2017). LisCVs are generated late (i.e., after 2-3 days of infection), when the bacteria cease to express ActA and polymerize actin, and are partially degradative; therefore, a subpopulation of bacteria survives in a quiescent state, raising the possibility of long-term persistence of *Listeria* in the liver parenchyma. Stages of persistence in hepatocytes have been described for other pathogens, such as hepatic viruses and parasites. For instance, *Plasmodium vivax* can enter a quiescent state within a parasitophorous vacuole to go undetected for years (Prudencio et al., 2006).

The objective of this study was to characterize the transcriptional response of the hepatocyte to long-term *Listeria* infection in order to identify a gene expression signature associated with intracellular bacterial persistence. We report the development of three robust cellular models to obtain a homogeneous population of hepatocytes hosting *Listeria* in the late LisCV stage. By comparing the *Listeria*-induced transcriptional pattern in these three different models, we found a common signature associated with *Listeria* persistence in the hepatocyte. Our data suggest that down-regulation of key hepatic innate immunity genes involved in the acute phase response (APR) may contribute to silent carriage of *Listeria* in the liver.

## Materials and Methods

### Bacterial Strains and Human Hepatocyte Cell Lines

We used *Listeria monocytogenes* laboratory strains EGDe and 10403S (Becavin et al., 2014) and a clinical isolate of the 4b serotype, CLIP80459 (Hain et al., 2012). Bacteria were grown on brain-heart infusion (BHI) medium at 37°C. The human hepatocellular carcinoma cell lines HepG2 (ATCC HB-8065) and Huh7 (CLS 300156) were grown in Dulbecco’s Modified Eagle Medium (DMEM, Gibco) supplemented with 2 mM L-glutamine (Sigma) and 10% fetal bovine serum (FBS, Sigma) at 37°C in a humidified 5% CO_2_ atmosphere and placed at 10% CO_2_ during infection assays.

### Isolation and Culture of Primary Mouse Hepatocytes

Primary mouse hepatocytes (PMH) were isolated from 8- to 10-week-old female C57BL/6 mice, by collagenase perfusion of the liver, as previously described (Fortier et al., 2017). Briefly, mice were anesthetized, and a midline laparotomy was performed. The inferior vena cava was perfused with a 0.05% collagenase solution (collagenase from *Clostridium histolyticum*, Sigma C5138). The portal vein was sectioned, and the solution allowed to flow through the liver. Upon liver digestion, hepatic cells were removed by mechanical dissociation, filtered through a sterile 70 μm cell strainer (BD Falcon), washed twice by centrifugation at 300 x g for 4 min. After a filtration step through a sterile 40 μm cell strainer (BD Falcon), cells were resuspended in serum-containing culture medium [DMEM, 10% FBS, 1% penicillin–streptomycin (Sigma); 100 μg/mL fungizone antimycotic B (Gibco)]. Cell count and viability were assessed by trypan blue exclusion. Cells were seeded in 6-well collagen-coated plates for 6 h at 37°C in a 5% CO_2_ atmosphere. After complete adhesion of the hepatocytes and washes to remove the dead cells, PMH were cultured at 37°C in a 5% CO_2_ atmosphere for 4-6 days before infection in serum-free hepatocyte culture medium [William’s E medium and GlutaMAX™ Supplement (Gibco); 100 U/ml penicillin/streptomycin, 0.5 μg/ml fungizone antimycotic B, 4 μg/ml insulin, 0.1% bovine serum albumin (BSA) and 25 nM dexamethasone (Sigma)] that was renewed daily.

### Antibodies and Reagents

The primary antibodies used in this study were anti-*L. monocytogenes* polyclonal rabbit antibody (BD Difco, 223001) and anti-human LAMP1 monoclonal mouse antibody (BD Bioscience, 555801). Fluorescent secondary antibodies were Alexa Fluor 488-conjugated goat anti-rabbit (Life Technologies) and Alexa Fluor Cy3-conjugated goat anti-mouse (Jackson ImmunoResearch Laboratories). Alexa fluor 647-conjugated phalloidin (Life Technologies) and Hoechst (Thermo Fisher Scientific) were used to label F-actin and nuclei, respectively. All recombinant human proteins were obtained in lyophilized form from R&D Systems and reconstituted according to the manufacturer’s instructions: IL-29/IFN-λ1 (1598-IL), IFN-β (8499-IF), interleukin IL-6 (206-IL) and IL-1β/IL-1F2 (201-LB).

### Bacterial Infections and Cytokine Treatment

HepG2 and Huh7 cells were seeded in 12- or 6-well plates in order to reach approximately 90% confluency on the day of infection. HepG2 cells were grown on collagen-coated wells or coverslips (type I collagen from rat tail, Sigma). Cells were counted using a hemocytometer (Neubauer-improved, Hausser Scientific) before infection to determine the multiplicity of infection (MOI). Inoculums were prepared in serum-free culture medium using bacteria grown overnight to stationary-phase and washed twice in PBS. Cells were infected as described in (Bierne et al., 2021) and subsequently incubated for the indicated times with culture media containing 25 μg/ml gentamicin to kill extracellular bacteria. To determine the number of intracellular bacteria at a given time-point post-infection (p.i.), cells were washed in serum-free culture medium and lysed in cold distilled water. Serial dilutions of cell lysates were plated on BHI agar and the number of intracellular bacteria was determined by counting colony-forming units (CFU) after 48 h incubation at 37°C. In parallel, host cells were enumerated following trypsin detachment and trypan-blue staining. When HepG2 and Huh7 infected and non-infected cells were stimulated with recombinant human cytokines, experiments were performed in 6-well plates and the specified concentration was obtained by adding 200 µl of culture medium containing 25 µg/ml gentamicin and the necessary volume of either the cytokine solution or a mock solution. PMH were infected using the same protocol as for the human cell lines, with the following differences: inoculum was prepared using William’s E medium and replaced after 1 h with hepatocyte culture medium depleted of penicillin/streptomycin and supplemented with 25 µg/ml gentamicin. Medium was renewed daily throughout the infection time course. Infected and non-infected cells were handled identically, all experiments were performed in triplicate and reproduced at least three times.

### Immunofluorescence Microscopy

Observation of intracellular *L. monocytogenes* by microscopy was performed as in (Bierne et al., 2021). Briefly, cells grown on 12 mm or 22 mm coverslips were rinsed in 1× phosphate-buffered saline (PBS) and fixed in 4% paraformaldehyde (PFA) in PBS for 30 minutes at room temperature. Cells were washed in PBS, permeabilized in 0.4% Triton X-100 in PBS, washed 3 times in PBS and incubated in blocking solution (2% bovine serum albumin (BSA) in PBS), before being processed for immunofluorescence, successively with the primary antibody solution, and secondary antibody solution containing Alexa fluor 647-conjugated phalloidin and Hoechst (in 2% BSA). Coverslips were mounted on glass slides using Fluoromount-G mounting medium (Interchim, Montlucon). Samples were analyzed with a Carl Zeiss AxioObserver.Z1 microscope equipped with 20× non-oil immersion or 40×, 63× and 100× oil immersion objectives connected to a CCD camera. Images were processed using ZEN (Carl Zeiss) or ImageJ.

### RNA Extraction, RNA-Sequencing, and Functional Gene Analysis

HepG2 or Huh7 cells were infected with strain EGDe or remained non-infected, in independent biological triplicates, and PMH isolated from the livers of two mice (n=3 per mouse) were infected with strain 10403S or remained non-infected. All infection assays were performed in 6-well plates (HepG2 and Huh7: 3 infected, 3 non-infected; PMH, 6 infected, 6 non-infected). RNA was extracted using the RNeasy Mini Kit (Qiagen) and genomic DNA was removed using TURBO DNA-free™ kit (Ambion), according to the manufacturer’s instructions. The integrity, purity and concentration of RNA samples was assessed on an RNA 6000 Nano chip using the Agilent 2100 electrophoresis Bioanalyzer. RNA Integrity Numbers (RIN) for all samples used for RNA sequencing (RNA-seq) library preparation was superior to 9.5. RNA-seq and data analysis procedures, as well functional gene analysis, and information on data repository, are detailed in the **Supplementary Material**. Each RNA-seq analysis was validated by RT-qPCR (see below), with *YWHAZ*, *PPIA*, or *Pdk1* used to normalize gene expression in infected relative to non-infected HepG2, HuH7 and PMH, respectively. Pearson correlation analysis was applied to the log2 FC of 14 significantly deregulated genes obtained by RNA-seq and RT-qPCR, as shown in the **Supplementary Material**, and indicates a very strong correlation.

### Reverse Transcription-Quantitative PCR

RNA was extracted from cultured cells and depleted for DNA, as described above. RNA concentration and purity were assessed using a NanoDrop spectrophotometer (Thermo Scientific). One microgram of total RNA was used for reverse transcription with the LunaScript™ RT SuperMix Kit (NEB). Quantitative Real-Time PCR was performed on StepOne Plus Real-Time PCR Systems (Applied Biosystems) using Luna^®^ Universal qPCR Master Mix (NEB) as specified by the supplier. Each 20 µl reaction was performed in triplicate. Target gene expression levels were normalized to an endogenous control gene whose expression stability was assessed using RefFinder (http://www.leonxie.com/referencegene.php) (Xie et al., 2012). PCR efficiencies were calculated to ensure equivalent amplification between target genes and endogenous control genes. Relative expression of target genes was calculated from cycle threshold (CT) values using the comparative CT (ΔΔCT) method with untreated or non-infected cells used as calibrator (reference) samples. Standard deviation of ΔCT values was determined according to the Applied Biosystems Guide to Performing Relative Quantitation of Gene Expression Using Real-Time Quantitative PCR (available at https://assets.thermofisher.com/TFS-Assets/LSG/manuals/cms_042380.pdf). A transcript was considered undetectable at CT ≥ 35; all negative controls in the absence of cDNA template generated CTs above this threshold. As IFN gene expression was absent in non-infected cells, this CT threshold was applied to all non-infected samples in order to allow the calculation of a relative expression value. Statistical significance of the difference in mean expression of genes from at least 3 experimental replicates was evaluated using the two-tailed paired-sample *t* test; a *p* value <0.05 was considered significant. Sequences of human and mouse gene specific primers are listed in **Supplementary Table S1**.

### ELISA Assays

Sandwich enzyme-linked immunosorbent assay (ELISA) kits were used to determine the concentrations of IFN-λ1 (Invitrogen, 88-7296), IFN-β (R&D Systems DIFNB0), and C3 (Abcam, ab108823) in the supernatants of infected and non-infected HepG2 and Huh7 cells, according to the respective manufacturers’ instructions. Conditioned media were collected at the indicated time-points p.i., centrifuged at 300 × g for 5 minutes at 4°C and the collected supernatants stored at -80°C. 50 μl of each sample supernatant and all standards were assayed in duplicate. For the C3 ELISA, conditioned media were diluted 100-fold in the diluent buffer according to the supplier’s protocol. Signals were detected using a Tecan Infinite 200 device (Tecan Trading AG, Switzerland) by reading the absorbance at 450 nm, from which readings at 570 nm were subtracted for wavelength correction. Statistical analyses were performed using Student’s two-tailed T-test on calculated concentrations.

### Protein Extraction and LC-MS/MS Analysis of Culture Supernatants

HepG2 and Huh7 cells in 6-well plates either remained non-infected or were infected with *L. monocytogenes* EGDe (MOI~1-5). After 72 h, cells were rinsed twice in serum-free medium and left for an additional 24 h in serum-free medium containing 25 µg/ml gentamicin. Conditioned media was then collected, supplemented with protease inhibitors mixture (Complete, EDTA-free, Roche), centrifuged (1000 × g, 5 min) and the supernatant concentrated ten times using an Amicon centrifugal filter unit (3K cut-off, Millipore). Cells were lysed in 500 ul of lysis buffer (50 mM Tris HCl pH 7.5, 150 mM NaCl, 1% Igepal, 0.1% SDS, 5% glycerol, supplemented with protease inhibitors cocktail) and incubated on ice (30 min). The cell lysate was cleared by centrifugation (14000 × g, 10 min, 4°C) with the supernatant assayed for protein concentration using the Bradford assay (Biorad). Protein concentration of cell lysates was used to normalize the amount of conditioned medium to concentrate between experimental conditions and replicates. Sample preparation for proteome analysis, liquid chromatography and Mass Spectrometry, as well as data analyses are detailed in the **Supplementary Material**.

## Results

### Establishment of Cellular Models of Long-Term *Listeria* Infection in Hepatocytes

To study the response of hepatocytes to long-term intracellular *Listeria* infection, we took a comparative approach using different cell models. However, prior to transcriptomic studies, it was essential to optimize protocols by testing different hepatocyte types, culture conditions, *Listeria* strains, and multiplicity of infection (MOI), in order to obtain populations of homogenously infected cells at 3 days post-infection (p.i.). We first selected the human hepatoblastoma cell line HepG2, widely exploited as an *in vitro* model of human hepatocytes and previously used to characterize the *Listeria* persistence stage (Kortebi et al., 2017). As these cells grow in clusters, leading to the formation of highly infected foci among predominantly uninfected islets, we seeded HepG2 cells on collagen-coated wells to allow the formation of a cell monolayer. Microscopic observation revealed that, for an infection at MOI~1-5 with the laboratory *Listeria* strain EGDe, collagen treatment was accompanied by a marked increase in bacterial intercellular spread during the 3-day course of infection (**Supplementary Figure S1A**), leading to LisCV formation at 72 h p.i. (**Supplementary Figure S1B**), as previously observed in conventionally grown HepG2 cells (Kortebi et al., 2017), but in a higher proportion of cells.

**Figure 1:**
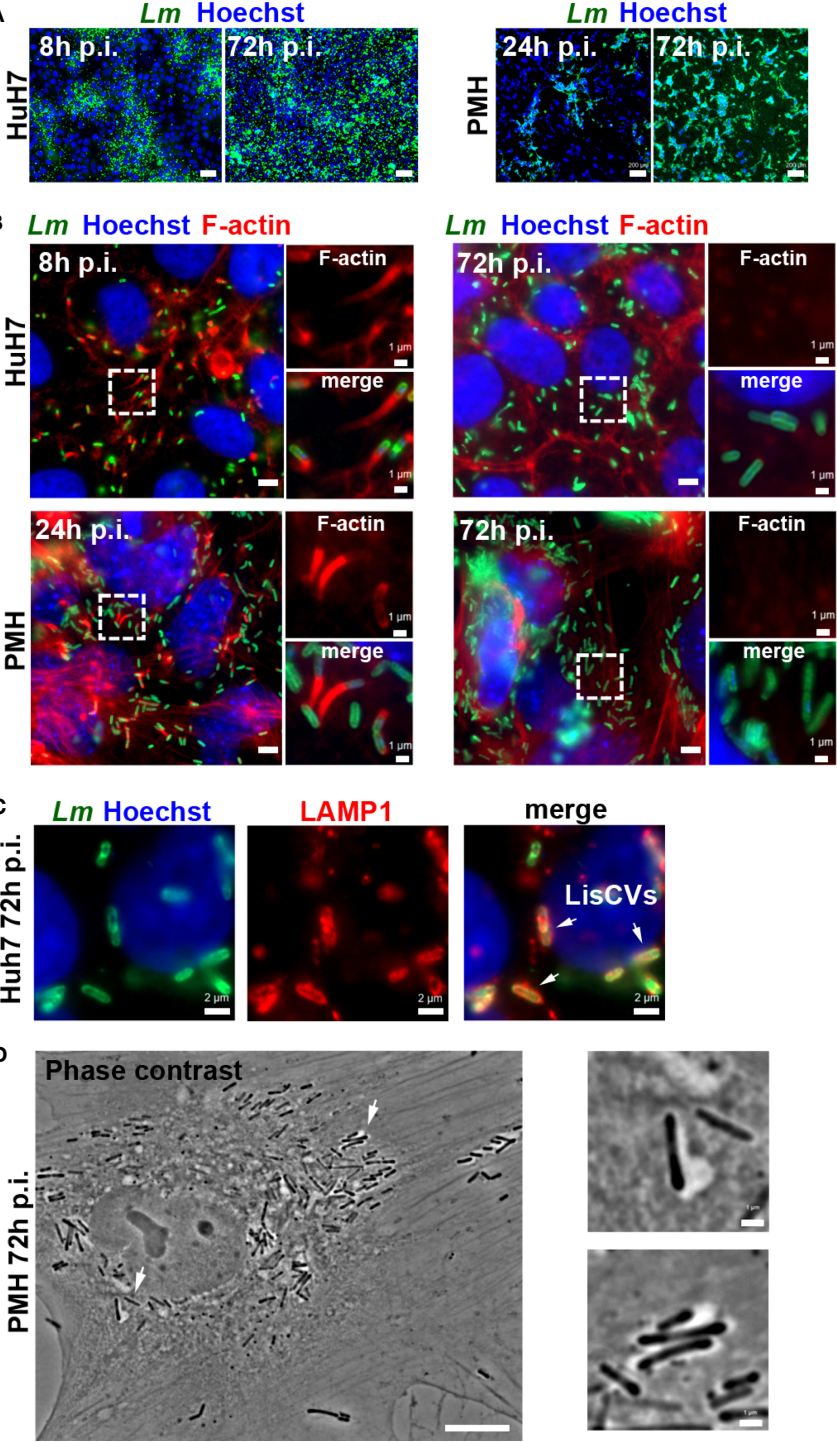
Optimization of hepatocyte culture systems for modeling persistent *Listeria* infection. Different cell seeding conditions, MOI and *Listeria* strains (EGDe or 10403S) were tested to obtain optimal long-term *Listeria* infection of HepG2 (see **Supplementary Figure S1**), Huh7 or PMH. Infected cells were examined at day 1 (d1) and at day 3 (d3) by immunofluorescence microscopy: representative examples under optimized conditions are shown. **(A)** Low magnification micrographs of Huh7 cells infected with EGDe strain (MOI=1-5) or PMH infected with 10403S strain (MOI=10) for the indicated time. Images are overlays of *Listeria* (green) and Hoechst (blue) signals (bars: 50 µm, Huh7, or 200 µm, PMH). **(B)** High magnification micrographs of infected Huh7 or PMH showing *Listeria* (green), F-actin (red) and Hoechst (blue) signals. Bars: 5 µm. Boxed regions enlarged on the right show F-actin (top) or merged signals (bottom), highlighting actin-positive bacteria at d1 and actin-negative bacteria at d3 (bars: 1 µm). **(C)** Micrographs of an infected Huh7 cell at d3, showing *Listeria* (green), LAMP1 (red) and Hoechst (blue) signals. Arrows indicate 3 examples of LisCVs. **(D)** Phase contrast image of an infected PMH at d3 (bars: 10 µm). Arrows indicate 2 examples of bacteria within vacuoles, shown at a higher magnification on the right (bars: 1 µm).

We also selected the hepatocellular carcinoma-derived cell line Huh7, which has been instrumental in exploring the mechanisms behind chronic hepatic viral infections (Hu et al., 2019; Todt et al., 2020). As opposed to HepG2, Huh7 naturally grow in monolayers and are highly permissive to *Listeria* cell-to-cell dissemination (**Figure 1A**). For the same MOI ~1-5 and *Listeria* strain (EGDe), the mean number of bacteria per infected cell at 72 h p.i. was on average ~10-fold higher in Huh7 cells than in HepG2 cells, as evaluated by Colony-Forming-Unit (CFU) and cell counts. Importantly, immunofluorescence experiments revealed that *Listeria* switched from polymerizing host actin at day 1 to the actin-free stage at day 3 (**Figure 1B**) accompanied by engulfment of bacteria into LAMP1-positive compartments (**Figure 1C**). Similar results were obtained with another commonly used laboratory strain, 10403S, as well as an epidemic clinical strain, CLIP80459 (**Supplementary Figure S2**). These results validate Huh7 as an additional model to study *Listeria* persistence in human hepatocytes.

As a third cell model, we chose primary hepatocytes, which could reveal cellular pathways potentially dysfunctional in carcinoma-derived cell lines. We previously showed that *Listeria* enters the persistence phase in primary human hepatocytes (PHH) (Kortebi et al., 2017), but we could not improve the infection protocol to use these cells for transcriptomic purposes, as they remained weakly infected with *Listeria*, regardless of the MOI or strain used. In particular, we found that thawing commercial PHH generated a significant viability issue, limiting the formation of intercellular junctions. We therefore used mouse hepatocytes, as they could be isolated fresh from animal livers immediately prior to infection. We found that freshly isolated primary mouse hepatocytes (PMH) were highly permissive to *Listeria* infection with strain 10403S at an MOI of 10 (**Figure 1A**). Furthermore, in PMH *Listeria* also shifted from the actin-dependent intercellular dissemination phase, at day 1, to the vacuolar persistence phase, at day 3 (**Figures 1B, D**). Overall, these results established that HepG2 and Huh7 cell lines and PMH are suitable models to study the hepatocyte response to persistent *Listeria* infection.

### Transcriptional Responses of Hepatocytes After a 3-Day *Listeria* Infection

**Figure 2:**
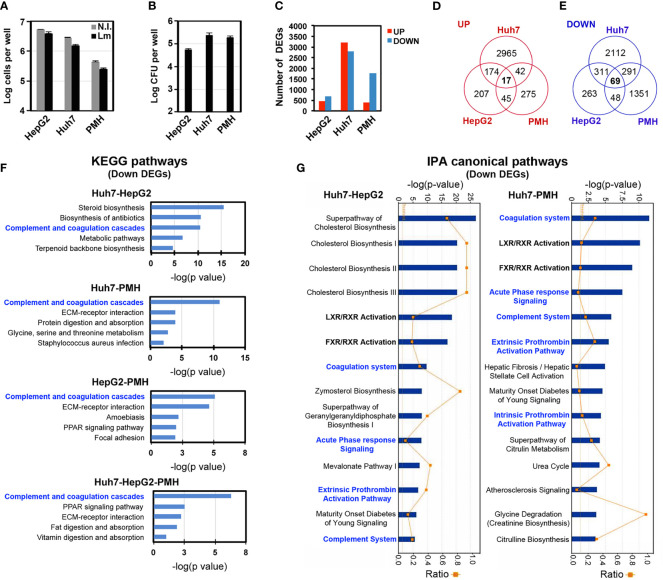
Transcriptional responses to long-term *Listeria* infection in hepatocytes. HepG2, Huh7 and PMH were infected with *Listeria* for 72 h. **(A)** Cell counts (log cells per well, mean ±SD, n=3). **(B)** Intracellular bacteria load expressed as log CFU per well (mean ±SD, n=3). **(C)** Number of significant DEGs upregulated (red bars) or downregulated (blue bars) in infected hepatocytes compared to non-infected hepatocytes (adj *p*<0.05; |log2 FC| > 0.5). **(D, E)** Venn diagram showing the intersection of upregulated **(D)** and downregulated **(E)** DEGs between HepG2, Huh7 and PMH datasets. **(F)** The top 5 most significant KEGG pathways associated with downregulated DEGs in each of the 4 overlapping DEG datasets. **(G)** The top 14 most significant IPA canonical pathways associated with the Huh7–HepG2 (left) and the Huh7–PMH (right) overlapping downregulated DEGs.

In order to identify host gene signatures specific to long-term *Listeria* infection in hepatocytes, we performed RNA-seq analysis on RNA extracted from HepG2, Huh7 or PMH infected for 72 h with *Listeria* strain EGDe (HepG2 and Huh7) or 10403S (PMH) and compared the gene expression profile to that of non-infected cells. The number of cells and intracellular bacteria were monitored in parallel to assess cell viability, revealing little cytotoxicity induced by infection (**Figure 2A**), and to compare intracellular bacterial loads between experiments and models (**Figure 2B**). Analysis of differentially expressed protein-coding genes between infected and non-infected cells (*p*<0.05 and log2 fold change (FC) >0.5 or <-0.5), herein termed “DEGs”, identified 1134 DEGs in HepG2 cells (443 upregulated and 691 downregulated, **Supplementary Tables S2**, **S3**), 5981 DEGs in Huh7 cells (3198 upregulated and 2783 downregulated, **Supplementary Tables S4**, **S5**) and 2138 DEGs in PMH (379 upregulated and 1759 downregulated, **Supplementary Tables S6**, **S7**) (**Figure 2C**). While intracellular bacterial loads were of the same range in all three models (on the order of 10^5^ CFU per well) (**Figure 2B**), we noted that the higher infectivity observed in Huh7 and PMH cells compared with HepG2 cells (7-fold and 4-fold, respectively) paralleled with a higher number of DEGs (5-fold and 2-fold respectively).

Functional analysis of upregulated DEGs revealed that interferon (IFN) responses were strongly activated in HepG2 and PMH cells, but not in Huh7 cells, after a 3-day infection with *Listeria* (see below). The 15 most highly upregulated DEGs in HepG2 cells included the interferon lambda 1 gene (*IFNL1*), as well as Interferon Stimulated Genes (ISGs) (i.e., *IFI44, RSAD2, IFI44L, OASL, IFIT2, OAS2, CH25H, CCL5, EPSTI1, CMPK2, IFIT1, IFIT3*) (**Supplementary Table S2**), as in PMH (i.e., *Ccl5, Ifit3b, Ifr7, Isg15, Zbp1, Ifi44, Ifit1, Ifit3, Oas3, Apol9b, Oas2l, Oas1h, Tgtp1, Rsad2*) (**Supplementary Table S6**). Indeed, 166 (37%) and 126 (33%) of the upregulated DEGs in HepG2 and PMH, respectively, were mapped as ISGs in the interferome database (Rusinova et al., 2013), of which 56 were common to both HepG2 and PMH datasets (**Supplementary Table S8**). In agreement with this, Gene Ontology of Biological Processes (GO-BP) and Kyoto Encyclopedia of Genes and Genomes (KEGG) pathway enrichment analysis showed that genes which were upregulated by infection in HepG2 and PMH cells significantly clustered in functions associated with viral infections and IFN responses (**Supplementary Table S9**). In contrast, upregulated DEGs in Huh7 cells were associated with entirely different biological processes and pathways, the most significant being “cell division” (GOBP) and “cell cycle*”* (KEGG) processes. This striking difference was also highlighted by the very limited overlap of upregulated DEGs between the three systems, with only 17 common genes (**Figure 2D**). 16 of these 17 genes were nonetheless categorized as ISGs (**Table 1** and **Supplementary Table S8**).

**Table 1:** Seventeen genes are upregulated by long-term *Listeria* infection in all hepatocyte models.

Gene Symbol	Gene Description	log2 FC		
		HepG2	Huh7	PMH
**CCL5**	C-C motif chemokine ligand 5	8.4	4.0	5.3
**CXCL10**	C-X-C motif chemokine ligand 10	5.7	1.2	2.1
**DDX60**	DExD/H-box helicase 60	8.3	1.2	2.6
**DHX58**	DExH-box helicase 58	1.5	2.3	2.8
**IFI44**	interferon induced protein 44	10.7	5.4	4.1
**IFIT2**	interferon induced protein with tetratricopeptide repeats 2	9.1	0.8	0.9
**ISG15**	ISG15 ubiquitin like modifier	3.7	0.6	4.3
**ISG20**	interferon stimulated exonuclease gene 20	1.3	0.6	0.7
**OAS2**	2’-5’-oligoadenylate synthetase 2	8.7	4.2	2.7
**OAS3**	2’-5’-oligoadenylate synthetase 3	3.2	1.5	3.7
**PMAIP1**	phorbol-12-myristate-13-acetate-induced protein 1	2.1	1.6	0.6
**RSAD2**	radical S-adenosyl methionine domain containing 2	10.5	4.3	3.6
SHLD3	shieldin complex subunit 3	0.7	1.0	0.5
**STAT1**	signal transducer and activator of transcription 1	2.2	0.8	1.8
**TRIM5**	tripartite motif containing 5	0.7	0.7	0.8
**ZC3HAV1**	zinc finger CCCH-type containing, antiviral 1	1.2	0.5	0.7
**ZNFX1**	zinc finger NFX1-type containing 1	1.2	0.9	0.9

Bolded gene symbols indicate genes that are classified as ISGs. Gray boxes indicate a log2 FC greater than 2 (i.e., 4-fold) in infected cells compared with non-infected cells.

With respect to downregulated DEGs, only 69 were common to all three datasets (**Figure 2E**). However, intersecting DEGs from only two datasets yielded a significant number of genes commonly downregulated (i.e., 380 DEGs common to HepG2 and Huh7 and 360 common to Huh7 and PMH). Functional analysis of each individual or intersecting dataset (**Supplementary Table S10**) highlighted the “coagulation and complement cascades” as one of the top 3 KEGG pathways most significantly perturbed by infection (**Figure 2F**). Ingenuity Pathway Analysis (IPA) also identified the “coagulation system*”* and “complement system*”* among the top 15 statistically significant canonical pathways altered by long-term *Listeria* infection (**Figure 2G**). Consistent with the fact that most complement proteins and several coagulation factors are acute phase proteins (APPs), the “acute phase response (APR) signaling*”* pathway was also significantly deregulated. It should be noted that in these pathways, the C1S complement component and SERPINA1 are encoded by a single gene in the human genome and multiple paralogs in the mouse genome. We thus included these two genes to the overlapping DEG datasets, leading to 71 genes (Huh7-HepG2-PMH, **Supplementary Table S11A**), 380 genes (Huh7-HepG2, **Supplementary Table S11B**) and 362 genes (Huh7-PMH, **Table S11C**), whose under-expression could be used as markers associated with *Listeria* persistent infection in hepatocytes (**Supplementary Table S11**).

In summary, the transcriptional signature of long-term *Listeria* infection is characterized by the robust activation of interferon response genes in HepG2 and PMH cells, and the inhibition of APR genes in all three hepatocyte models, with the downregulation of a large number of genes involved in the complement and coagulation cascades.

### Different Interferon Responses to *Listeria* Infection in HepG2, Huh7, and PMH Cells

**Figure 3:**
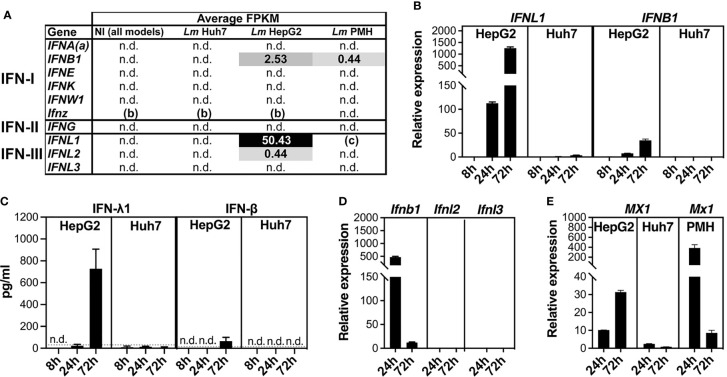
*Listeria* infection triggers interferon responses in HepG2 and PMH, but not in Huh7 cells. **(A)** IFN gene expression in non-infected (NI) and infected (*Lm* EGDe or 10403S) HepG2, Huh7 and PMH cells at 72 h p.i., assessed by mean FPKM for each type I, II, or III IFN gene. (a) IFNA represents all genes coding for IFN-α subtypes; (b) *Ifnz* is present in the mouse but not the human genome; (c) *Ifnl1* is a pseudogene in the mouse genome. “n.d.”, non detectable (FPKM < 0.3, the background level). **(B)** RT-qPCR analysis of *IFNL1* and *IFNB1* transcript levels in EGDe-infected HepG2 and Huh7 cells at 8 h, 24 h, and 72 h p.i., relative to NI cells, and normalized to *YWHAZ* and *PPIA* for HepG2 and Huh7, respectively. **(C)** ELISA quantification of IFN-λ1 (left) and IFN-β (right) protein secreted by EGDe-infected HepG2 or Huh7 cells. Conditioned media from EGDe-infected or NI cells was sampled at 8 h, 24 h, and 72 h p.i. Dotted grey lines: limit of quantification; “n.d.”: below the limit of detection (LoD). All NI cell conditioned media sampled concurrently with infected samples were below the LoD (not shown). **(D)** RT-qPCR analysis of *Ifnb, Ifnl2* and *Ifnl3* transcript levels in 10403S-infected PMH at 24 h and 72 h p.i., expressed relative to NI cells, and normalized to *Pdk3* and *Pdk1* for 24 h and 72 h time points, respectively. **(E)** RT-qPCR analysis of *MX1* transcript levels in EGDe-infected HepG2 or Huh7 cells, and *Mx1* in 10403S-infected PMH, at 24 h and 72 h p.i., relative to NI cells, and normalized to *YWHAZ*, *PPIA*, and *Pdk3/Pdk1* for HepG2, Huh7, and PMH respectively. Values represent mean ± SD (n=3).

The *Listeria*-induced interferon responses in HepG2 and PMH, strikingly absent in Huh7, prompted us to further examine the expression of IFN genes. We first analyzed the RNA-seq data to have information about the absolute expression levels (fragments per kilobase of transcript per million mapped reads, FPKM) of IFN transcripts coding for either type I IFNs (IFN-I: IFN-α subtypes, IFN-β, -ϵ, -κ, -δ, -ζ, -ω), type II IFN (IFN-II: IFN-γ), or type III IFNs (IFN-III: IFN-λ1, -λ2, -λ3). A threshold FPKM value of 0.3 was applied to define detectable expression above background, as previously described (Ramskold et al., 2009). No IFN gene transcripts were detected in non-infected samples of any of the three models, nor in infected Huh7 samples (**Figure 3A**). In contrast, 3-day *Listeria* infection, triggered the expression of *IFNL1* (coding IFN-λ1*)* and, to a lesser extent, *IFNB1* (coding IFN-β) and *IFNL2* (coding IFN-λ2) transcripts in HepG2 cells, while low levels of *IFNB1* transcripts were detected in infected PMH.

Reverse transcription-quantitative PCR (RT-qPCR) was then used to compare IFN gene expression at 72 h p.i. with that of earlier infection time points, in order to determine the time course and amplitude of IFN gene expression in infected hepatocytes. In HepG2 hepatocytes, *Listeria* infection induced *IFNL1* and *IFNB1* expression at 24 h p.i., and more significantly at 72 h p.i. (10-fold more for *IFNL1* and 4-fold more for *IFNB1)*, but not at 8 h p.i. (**Figure 3B**). In addition, *IFNL1* expression was approximately 20-fold higher than that of *IFNB1*. In Huh7 cells, consistent with FKPM values, *Listeria* infection did not induce expression of any of these genes at any time point. ELISA assays confirmed the major production of IFN-λ1 and minor production of IFN-β proteins in response to *Listeria* infection in HepG2, with highest amounts at 72 h p.i., and the absence of both IFNs in Huh7 (**Figure 3C**). In PMH, quantification of *Ifnb1, Ifnl2*, and *Ifnl3* transcript levels [murine *Ifnl1* is a pseudogene (Hermant et al., 2014)] showed that infection elicited the expression of only *Ifnb1*, which was 40 times higher at 24 h than at 72 h p.i. (**Figure 3D**). The expression profile of *MX1*, an ISG known to be induced by both IFN-I and IFN-III, followed the expression profile of IFN genes in HepG2 and PMH and was not significantly induced in Huh7-infected cells (**Figure 3E**). We conclude that HepG2 and PMH both induce IFN genes when exposed to *Listeria*, but exhibit differences, with an early activation of IFN-I in PMH and a late activation of IFN-I/III – essentially *IFNL1 *– in HepG2 cells. We also observed the Huh7 cell line to be defective in the signaling pathway leading to IFN production in the context of *Listeria* infection, as previously noted (Odendall et al., 2014).

However, Huh7 cells are known to have functional IFN receptors and can thus mount effective interferon secondary responses (Bolen et al., 2014; Odendall et al., 2014). We investigated whether the important bacterial burden observed in Huh7 cells could be the result of their defect in IFN signaling, by restoring functional responses using recombinant IFNs. Huh7 cells were infected with the EGDe strain for 24 h and treated daily with either IFN-β (3 ng/ml; ~800 IU/ml) or IFN-λ1 (100 ng/ml) for an additional 48 h. IFN treatment activated the expression of ISGs (*IFIT1, IFI6* and *MX1*), but did not result in a reduction in the number of intracellular bacteria at 72 h p.i. compared to untreated cells (**Supplementary Figure S3**).

Taken together, these results indicate that HepG2, Huh7 and PMH cells differ in their ability to mount interferon responses upon long-term *Listeria* infection, but interferon responses do not seem to affect the *Listeria* burden within hepatocytes.

### Long-Term *Listeria* Infection in Hepatocytes Downregulates Genes of the Complement and Coagulation Cascades

**Figure 4:**
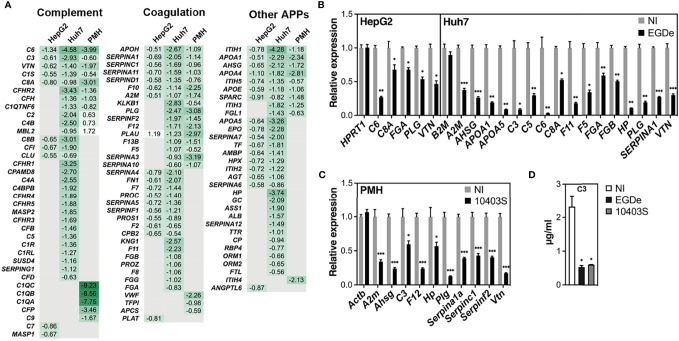
Long-term *Listeria* infection downregulates expression of APP genes in hepatocytes. **(A)** Heatmap displaying log2 FC of APP genes significantly deregulated in infected cells compared to non-infected (NI) cells. APPs are classified according to their role in either the complement or coagulation systems or other functions. Gene symbols are indicated (corresponding gene names can be found in **Supplementary Tables S3, S5, S7**). Human *C1S* and *SERPINA1* have several murine orthologues; FC for murine *C1s1* and *Serpina1a* are shown. **(B, C)** RT-qPCR analysis of representative genes in **(B)** EGDe-infected HepG2 or Huh7 cells, and **(C)** 10403S-infected PMH, relative to NI cells. A non-differentially regulated control gene (*HPRT1* for Huh7, *B2M* for HepG2, and *Actb* for PMH) is included for each set. **(D)** C3 protein concentration in the conditioned media of NI or infected Huh7 cells at 72 h p.i. was measured by ELISA. All values represent mean ± SD (n=3). Statistical significance, Student’s *t* test (*p < 0.05, **p < 0.01, ***p < 0.001).

Given the important roles of APP and other effectors of the complement and coagulation systems in immunity and host defense against pathogens (Merle et al., 2015; Antoniak, 2018; Ermert et al., 2019; Reis et al., 2019), dysregulation of these processes may have a strong impact on the outcome of *Listeria* infection. We thus explored further the nature of the downregulated DEGs associated with these innate immunity and hemostasis pathways. IPA and KEGG analysis, combined with a thorough literature search, identified 91, 44, and 43 APP genes which were downregulated by long-term *Listeria* infection in Huh7, HepG2, and PMH cells, respectively (**Figure 4A**), 19 of which were common to all three models. RT-qPCR was used to validate the downregulation of representative genes in HepG2, Huh7 cells (**Figure 4B**) and PMH (**Figure 4C**). The same downregulation was observed in Huh7 cells infected with *Listeria* strain 10403S or epidemic strain CLIP80459, compared to EGDe, demonstrating that the inhibitory effect was not strain-dependent (**Supplementary Figure S4**).

**Figure 5:**
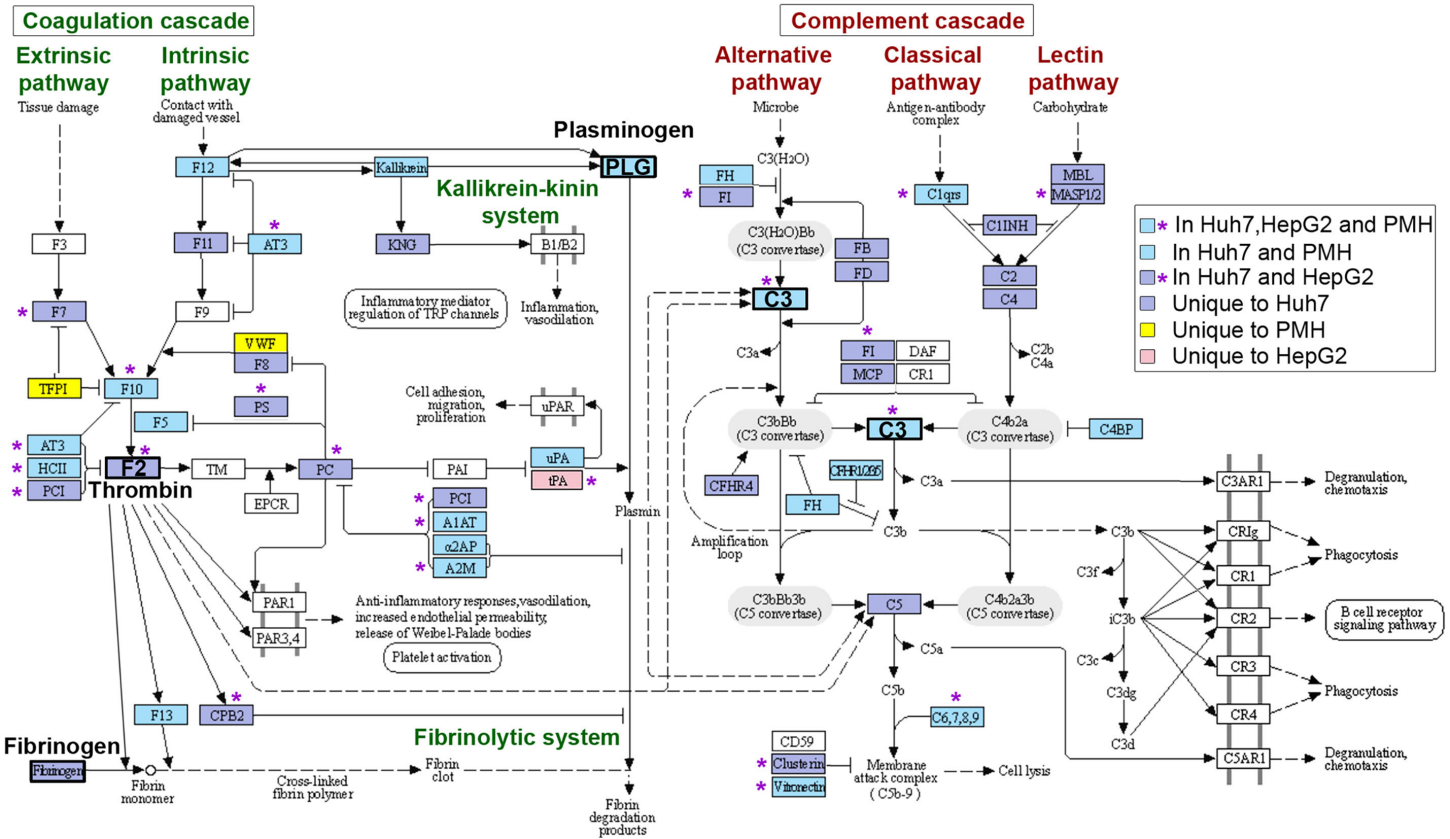
Mapping of APP genes downregulated upon long-term *Listeria* infection on the “complement and coagulation cascade” KEGG pathway. Colored boxes and purple stars indicate components of the cascades specifically altered in three, two or one hepatocyte model (as shown by the legend on the right). The hsa04610 KEGG pathway was adapted to underline different pathways of the coagulation cascade and fibrinolytic system (left) or complement cascade (right). Key components are highlighted in bold: Thrombin (*F2*), Fibrinogen (*FGA, FGB, FGG*), Plasminogen (*PLG*) and complement C3 (*C3*).

Downregulated APP genes unique to HepG2, Huh7 or PMH, or common to at least two models, were mapped onto the KEGG “complement and coagulation cascades” pathway (**Figure 5**). These complex cascades of tightly regulated proteolytic events act in crosstalk (Amara et al., 2008). The complement system can be activated by either the classical, lectin or the alternative pathway, which all converge to trigger the cleavage of a central component, the C3 protein and generate the same effector molecules. Strikingly, long-term infection with *Listeria* in Huh7 cells led to the downregulation of 30 genes, either involved in the complement cascade or acting as complement regulators (**Figure 4A**). Eight of these genes were similarly inhibited by *Listeria* infection in PMH: they encode (*i*) the activating enzyme C1S of the C1 complex, (*ii*) the central component C3, (*iii*) the membrane-attack proteins C6 and C8A, and (*iv*) four complement regulators (C1QTNF6, CFH, CFHR2, VTN) (**Figure 5**). Expression of genes encoding effectors of the blood coagulation and fibrinolysis systems were also altered by *Listeria* infection. The coagulation system includes the coagulation cascade and parallel regulatory systems that lead to a complex interplay of reactions resulting in the conversion of soluble fibrinogen to insoluble fibrin strands. The coagulation cascade is traditionally classified into the intrinsic and extrinsic pathways, both of which converge on Factor X activation, generation of thrombin, and subsequently fibrin to form fibrin clots. The fibrinolytic system (or plasminogen-plasmin system) is required for fibrin degradation and blood clot dissolution (**Figure 5**). Genes belonging either to extrinsic and intrinsic coagulation cascade, kallikrein-kinin system, fibrinolytic system and regulatory pathways, were widely downregulated upon long-term *Listeria* infection in Huh7 and PMH cells (32 and 19 DEGs, respectively), 16 of which were common to both hepatocyte models (**Figure 4A**). In particular, infection led to the repression of genes coding for (*i*) the coagulation factors F12, F10, F5, F13, and thrombin (F2), the enzyme responsible for the conversion of fibrinogen to fibrin, (*ii*) Kallikrein B1 (KLKB1) of the kallikrein-kinin system, a metabolic cascade resulting in the release of vasoactive kinins, (*iii*) plasminogen (PLG) and urokinase plasminogen activator (PLAU or u-PA) in the fibrinolytic system, (*iv*) a set of serpin proteases with regulatory functions (SERPINA1/α1-antitrypsin, SERPINA3/α1-antichymotrypsin, SERPINA10/Protein Z-dependent protease inhibitor, SERPINA11, SERPINC1/antithrombin, SERPIND1/heparin cofactor II, SERPINF2/α2-antiplasmin) and (*v*) other regulators (A2M/α2 macroglobulin, APOH/apolipoprotein H) (**Figure 5**). APP genes involved in other various biological processes were also downregulated in infected hepatocytes. In particular, common to Huh7 and PMH cells were 10 APPs contributing to the regulation of lipid metabolism (AHSG/Fetuin-A, APOA1, APOA4, APOA5, APOE, SPARC and ITIH5) or other functions (ITIH1, ITIH3, FGL1) (**Figure 4A**). The downregulation of complement and coagulation gene expression in infected hepatocytes compared to the basal level of expression in non-infected cells reflects the capacity of long-term *Listeria* infection to modify the hepatic innate immune landscape.

### Long-Term *Listeria* Infection Reduces the Amount of Secreted APPs

We next examined whether the decrease in APP mRNA levels induced by long-term *Listeria* infection translated into a reduced abundance of secreted APPs. We first quantified the concentration of the C3 protein in cellular supernatants by ELISA. In agreement with mRNA expression data, a significant reduction in total C3 levels was observed in the conditioned media recovered from infected Huh7 and HepG2 cells, compared to non-infected cells (**Figure 4D** **)**. We then performed a label-free MS quantitative proteomic analysis to explore the composition of the secretome of infected cells. The quantification of proteins in the conditioned media of long-term infected HepG2 or Huh7 cells compared to that of non-infected cells identified a significant decrease in the abundance of 42 and 153 proteins, respectively. Intersecting the secretome of each cell line with its corresponding transcriptome revealed a strong overlap, with 25 and 82 proteins that were downregulated at both the proteomic and transcriptomic level in HepG2 and Huh7, respectively (**Supplementary Table S12**), of which more than half were APPs (**Table 2**). These results suggest that long-term *Listeria* infection in hepatocytes alters APP secretion at a transcriptional level.

**Table 2:** Long-term *Listeria* infection decreases the abundance of APP in the secretome of HepG2 and Huh7 hepatocytes.

Uniprot accession	Gene symbol	Protein name	HepG2		Huh7 (a)	
			log2 Ratio	*p*-adj	log2 Ratio	*p*-adj
**Complement**						
P01024	C3	complement C3	-0.77	3.8E-19	-3.25	8.8E-59
P01031	C5	complement C5			-2.58	1.1E-16
P0C0L4	C4A	complement C4A			<-4.00	7.7E-15
P0C0L5	C4B	complement C4B			<-4.00	7.7E-15
P00751	CFB	complement factor B			-3.29	1.8E-09
P04004	VTN	vitronectin	-1.32	6.4E-03	-2.03	2.7E-06
P05155	SERPING1	C1-inhibitor			-4.09	1.6E-04
P06681	C2	complement C2			<-1.87	3.9E-04
P08603	CFH	complement factor H			-2.70	7.1E-18
Q03591	CFHR1	complement factor H related 1			<-2.74	1.3E-06
P10909	CLU	clusterin	-0.98	8.5E-03	-1.37	2.7E-04
P05156	CFI	complement factor I	-1.00	3.0E-03	<-1.87	3.9E-04
**Coagulation**						
P02751	FN1	fibronectin 1			-1.97	2.4E-28
P01023	A2M	alpha-2-macroglobulin	-0.90	1.7E-19	-2.08	8.8E-24
P00747	PLG	plasminogen			-4.55	1.9E-22
P01009	SERPINA1	alpha-1-antitryppsin	-0.80	4.1E-07	-2.06	5.2E-17
P01042	KNG1	kininogen 1			-3.91	5.5E-10
P02749	APOH	apolipoprotein H	-0.75	2.8E-03	-3.58	5.9E-10
P36955	SERPINF1	pigment epithelium-derived factor	-0.95	9.2E-06	-2.27	1.2E-07
P08697	SERPINF2	alpha-2-antiplasmin			-1.93	2.6E-06
P01008	SERPINC1	antithrombin			-1.97	6.2E-06
P05154	SERPINA5	plasminogen activator inhibitor-3	-0.98	9.3E-03	-2.37	8.8E-05
P05546	SERPIND1	heparin cofactor 2	-0.95	9.0E-03	-2.04	9.5E-05
P29622	SERPINA4	kallikrein inhibitor			<-1.58	1.5E-03
Q86U17	SERPINA11	serpin family A member 11	-1.23	2.4E-03	<-1.00	8.7E-03
P00748	F12	coagulation factor XII			-4.17	8.8E-05
P00734	F2	coagulation factor II, thrombin			-1.45	2.9E-04
P12259	F5	coagulation factor V			-3.91	5.0E-04
P02675	FGB	fibrinogen beta chain			-1.58	2.2E-03
**Other APPs**						
P02787	TF	transferrin	-0.65	7.0E-10	-1.43	1.6E-30
P02768	ALB	albumin			-1.22	2.4E-25
P02647	APOA1	apolipoprotein A1	-0.98	1.9E-06	-3.50	3.2E-22
P02760	AMBP	alpha-1-microglobulin/bikunin precursor	-0.89	2.4E-05	-2.12	1.4E-13
P02774	GC	GC, vitamin D binding protein			-5.09	4.8E-09
P19823	ITIH2	inter-alpha-trypsin inhibitor heavy chain 2	-0.86	4.4E-08	-1.15	1.4E-07
P01019	AGT	angiotensinogen			-1.76	2.2E-07
P02649	APOE	apolipoprotein E	-0.67	1.9E-03	-1.20	8.5E-07
P02765	AHSG	alpha 2-HS glycoprotein	-0.62	2.8E-03	-1.28	1.1E-05
P02753	RBP4	retinol binding protein 4			-1.24	8.0E-05
P00738	HP	haptoglobin			<-1.42	2.7E-03
P00450	CP	ceruloplasmin			-3.49	2.9E-11
Q08830	FGL1	fibrinogen like 1			-3.00	1.5E-03

A label-free quantitative proteomic approach was used to identify proteins differentially abundant in the conditioned medium of infected (I) compared to non-infected (NI) hepatocytes in 3 independent experiments. The relative abundance of individual proteins was assessed by significant differences in spectral counts (SC) between the two conditions (*p-*adj <0.01, see **Supplementary Material**). Proteins with significant decreases, expressed as log2 (I/NI) SC ratio, were then selected on the basis of a decrease in RNA level of the corresponding gene in the transcriptome dataset, resulting in the identification of 25 and 82 proteins in HepG2 and HuH7, respectively (see **Supplementary Table S12**), of which 17 and 41 were APPs. (a) For eight proteins with SC=0 in the Huh7 infected condition, an arbitrary value of SC=1 was used to allow the calculation of a log2 ratio (in this case, the value is preceded by "<").

### Long-Term *Listeria* Infection Impairs the Cytokine-Driven Expression of APP Genes

**Figure 6:**
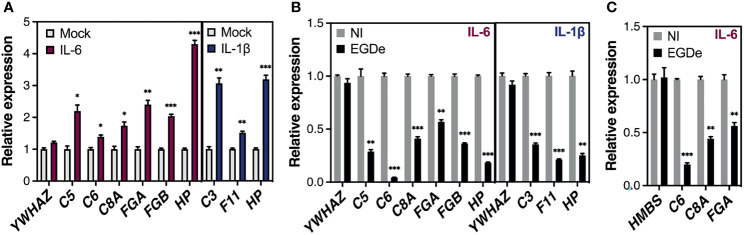
Long-term *Listeria* infection reduces cytokine-driven expression of APP genes in human hepatocytes. **(A)** Huh7 cells were stimulated with IL-6 (50 ng/mL) or IL-1β (10 ng/mL) for 24 h. Gene expression was analyzed by RT-qPCR and is expressed as fold-induction relative to mock-stimulated cells; IL-6 induced the expression of *C5*, *C6*, *C8A, FGA*, *FGB* and *HP* genes, while IL-1β induced *C3*, *F11* and *HP* genes. **(B, C)** Effect of infection on cytokine-induced APP gene expression in Huh7 **(B)** and HepG2 cells **(C).** Cells were infected with strain EGDe (MOI~1-5) for 48 h or remained non infected (NI). IL-6 (50 ng/mL) or IL-1β (10 ng/mL) (Huh7) or IL-6 (25 ng/mL) (HepG2) was added to infected and NI cells and transcript levels relative to stimulated NI cells were quantified by RT-qPCR 24 h later (at 72 h p.i., i.e. 24 h post-stimulation). A control gene whose expression was affected neither by infection nor cytokine stimulation is included. **(B)** In Huh7 cells, the control gene was *YWHAZ* and gene expression was normalized to *PPIA*; **(C)** In HepG2 cells, the control gene was *HMBS* and gene expression was normalized to *YWHAZ*. All values represent mean ± SD (n=3). Statistical significance, Student’s *t* test (*p < 0.05, **p < 0.01, ***p < 0.001).

Given their important physiological functions, most APPs are produced at a basal constitutive level by hepatocytes. However, their amounts rapidly change in response to pro-inflammatory cytokines released by innate immune cells during the course of an infection, with interleukin-6 (IL-6) and interleukin-1 (IL-1)-type cytokines as the leading inducers (Zhou et al., 2016). As long-term *Listeria* infection reduced basal levels of APP gene expression, we sought to evaluate the effect of long-term *Listeria* infection on cytokine-induced APP gene expression. Since IL-6 and IL-1β produce selective gene induction, we first applied these cytokines to non-infected Huh7 cells, for 24 h, to identify representative IL-6- and IL-1β-specific APPs [also known as type I and type II APPs, respectively (Baumann and Gauldie, 1994)]. IL-6 induced the expression of *C5*, *C6*, *C8A, FGA*, *FGB* and *HP* genes, while IL-1β induced *C3*, *F11* and *HP* genes (**Figure 6A**). We subsequently examined the effect of *Listeria* infection on the cytokine-induced expression of these genes. Our results showed that upon inflammatory cytokine stimulation, the mRNA abundance of APPs was strongly reduced in Huh7-infected cells, when compared to non-infected cells, indicating that infection inhibits the inflammatory signaling pathway (**Figure 6B**). We confirmed this effect in HepG2 cells for the APPs *C6* and *FGA*, following stimulation with IL-6 (**Figure 6C**). These results reveal that long-term *Listeria* infection not only represses the constitutive expression of APP genes, but also counteracts the transcriptional stimulation induced by inflammatory stimuli.

### Long-Term *Listeria* Infection Downregulates Genes of LXR-RXR, FXR-RXR, and Cholesterol Metabolism-Associated Pathways

**Figure 7:**
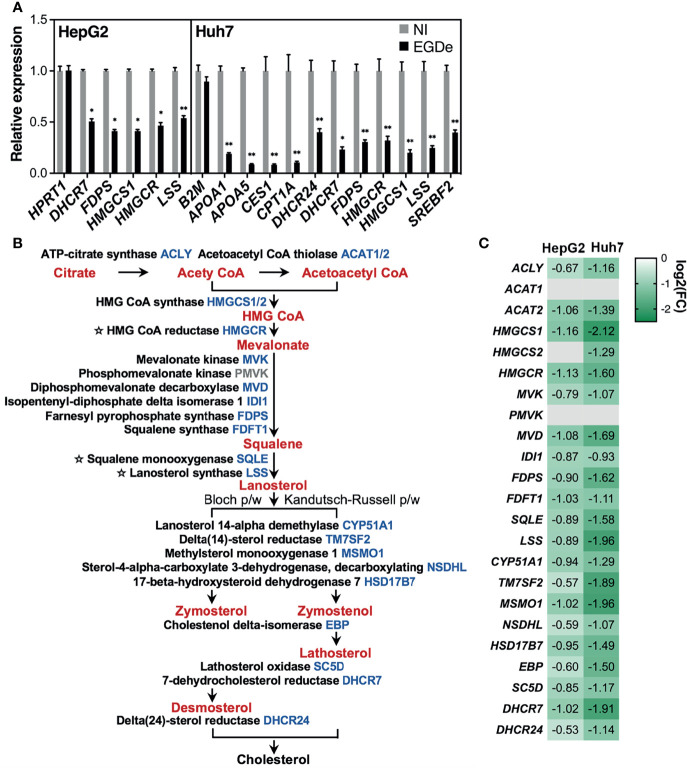
Long-term *Listeria* infection downregulates almost all genes of the cholesterol biosynthesis pathway in both human HepG2 and Huh7 cells. **(A)** RT-qPCR analysis of transcript levels of representative genes involved in cholesterol metabolism and homeostasis in EGDe-infected HepG2 and Huh7 cells, relative to non-infected (NI) cells. Expression was normalized to *YWHAZ* or *PPIA* for HepG2 and Huh7 cells, respectively, and a non-differentially expressed control gene (*HPRT1* for HepG2, *B2M* for Huh7) is included. Values represent mean ± SD (n=3). Statistical significance, Student’s *t* test (*p < 0.05, **p < 0.01). **(B)** Diagram illustrating the *de novo* cholesterol biosynthesis pathway. Enzyme names are in black and their corresponding gene symbols are blue when downregulated in infected cells, otherwise they are gray. Rate limiting enzymes are preceded by a star. Intermediary molecules are shown in red. Lanosterol can be transformed into cholesterol *via* two different routes: the Bloch pathway (left) and the Kandutsch-Russell pathway (right). **(C)** Heat map showing the log2 FC obtained by RNA-seq in HepG2 and Huh7 infected versus NI cells, for each of the cholesterol synthesis enzyme coding genes listed in **(B)**.

IPA analysis of genes which were downregulated after 3-day *Listeria* infection, either in both human hepatocyte models, or both human Huh7 and PMH models, also identified pathways associated with ligand-dependent nuclear receptors liver X receptor/retinoid X receptor (LXR/RXR) and farnesoid X receptor/retinoid X receptor (FXR/RXR) (**Figure 2G**). LXR and FXR play a critical role in the regulation of metabolism, particularly cholesterol, fatty acid, bile acid and carbohydrate metabolism (Ding et al., 2014). Of note, several genes associated with these pathways encoded APPs (e.g., *AHSG, APOA1, APOA4, APOC1, APOC3, APOE, APOH* and *SERPINF2*), in line with the notion that LXR and FXR act in crosstalk with other transcription factors to control expression of a set of APP genes (Odom et al., 2004; Wollam and Antebi, 2011; Yin et al., 2011). This was confirmed at the protein level, as infection decreased the amount of secreted AHSG and apolipoproteins APOA1, APOE and APOH, in both HepG2 and Huh7 conditioned media, as well as of APOB, APOC3 and SERPINF2 in Huh7 (**Supplementary Table S12**). In addition, genes involved in bile acid and cholesterol biosynthesis pathways, namely bile acid-CoA:amino acid N-acyltransferase (*BAAT*) and 3-hydroxy-3-methylglutaryl-CoA synthase 2 (*HMGCS2*), respectively, and several apolipoprotein genes (*APOA1, APOA4, APOC1, APOE, APOH*) were downregulated PMH as in Huh7 cells (**Supplementary Table S11**). It was particularly striking that cholesterol metabolism-related pathways were the most highly significantly altered pathways in HepG2 and Huh7 transcriptome datasets, as shown by KEGG (**Figure 3G**) and IPA enrichment analysis (**Figure 3F**). RT-qPCR validated the downregulation of representative genes (**Figure 7A**). As for APP genes, this inhibitory effect was not strain-dependent (**Supplementary Figure S4**). In both HepG2 and HuH7 hepatocytes, nearly all the genes of the cholesterol biosynthesis pathway were downregulated, including the genes encoding the rate limiting enzymes 3-hydroxy-3-methylglutaryl coenzyme A reductase (*HMGCR*), squalene epoxidase (*SQLE*), and lanosterol synthase (*LSS*) (**Figures 7B** **, C** **).** In Huh7, several genes of the classical bile acid (BA) synthesis pathway were also downregulated, including the rate limiting enzyme cytochrome P450 family 7 subfamily A member 1 (*CYP7A1*). Together, these results suggest that long-term *Listeria* infection disturbs the expression of cholesterogenic-, lipogenic- and bile acid-associated genes.

### Upstream Regulator Analysis of Gene Networks Perturbed by Long-Term *Listeria* Infection

To propose mechanistic hypotheses that could explain the inhibitory effect of *Listeria* infection on APP and lipid metabolism gene expression we used the Ingenuity Upstream Regulator Analysis (URA) analytical algorithm to predict host transcriptional regulators targeted by infection. This analysis identified several transcription factors (TFs), ligand-dependent nuclear receptors, as well as epigenetic factors, as possible regulators underlying infection-orchestrated transcriptional modulation (**Supplementary Table S13**). URA performed on the network of genes that were either deregulated by infection in Huh7 and PMH models, or in human Huh7 and HepG2 models, identified extensive overlap between the transcriptional regulators coordinating the observed patterns of gene dysregulation: the hepatocyte nuclear factors HNF1α and HNF4α, the ligand-dependent nuclear receptors PPARα, LXRβ, RXRα and RORC, and P53. The Huh7 and PMH models also revealed the possible involvement of CEBPA (CCAAT/enhancer binding protein alpha), a TF known to regulate APP expression with hepatocyte nuclear factors, as well as the epigenetic regulators EZH2, EP300, and SMARCB1. The sterol regulatory element-related transcription factors SREBF1 (or SREBP1) and SREBF2 (or SREBP2), on the other hand, were specific to Huh7 and HepG2 models, consistent with their function as master transcriptional regulators of cholesterol homeostasis (Shimano and Sato, 2017). Interestingly, the epigenetic regulator SIRT2, previously associated with *Listeria* infection (Eskandarian et al., 2013), was predicted to regulate the expression of ten infection-dysregulated genes involved in the cholesterol biosynthetic pathway (*ACLY, DHCR7, FDFT1, HMGCR, IDI1, LSS, MVD, MVK, SC5D, SQLE*) (**Supplementary Table S13**) (**Figure 7B**). Together, these analyses provide insights into transcriptional regulators that may orchestrate gene dysregulation mediated by long-term *Listeria* infection of hepatocytes and impact the intracellular persistence phase.

## Discussion

**Figure 8:**
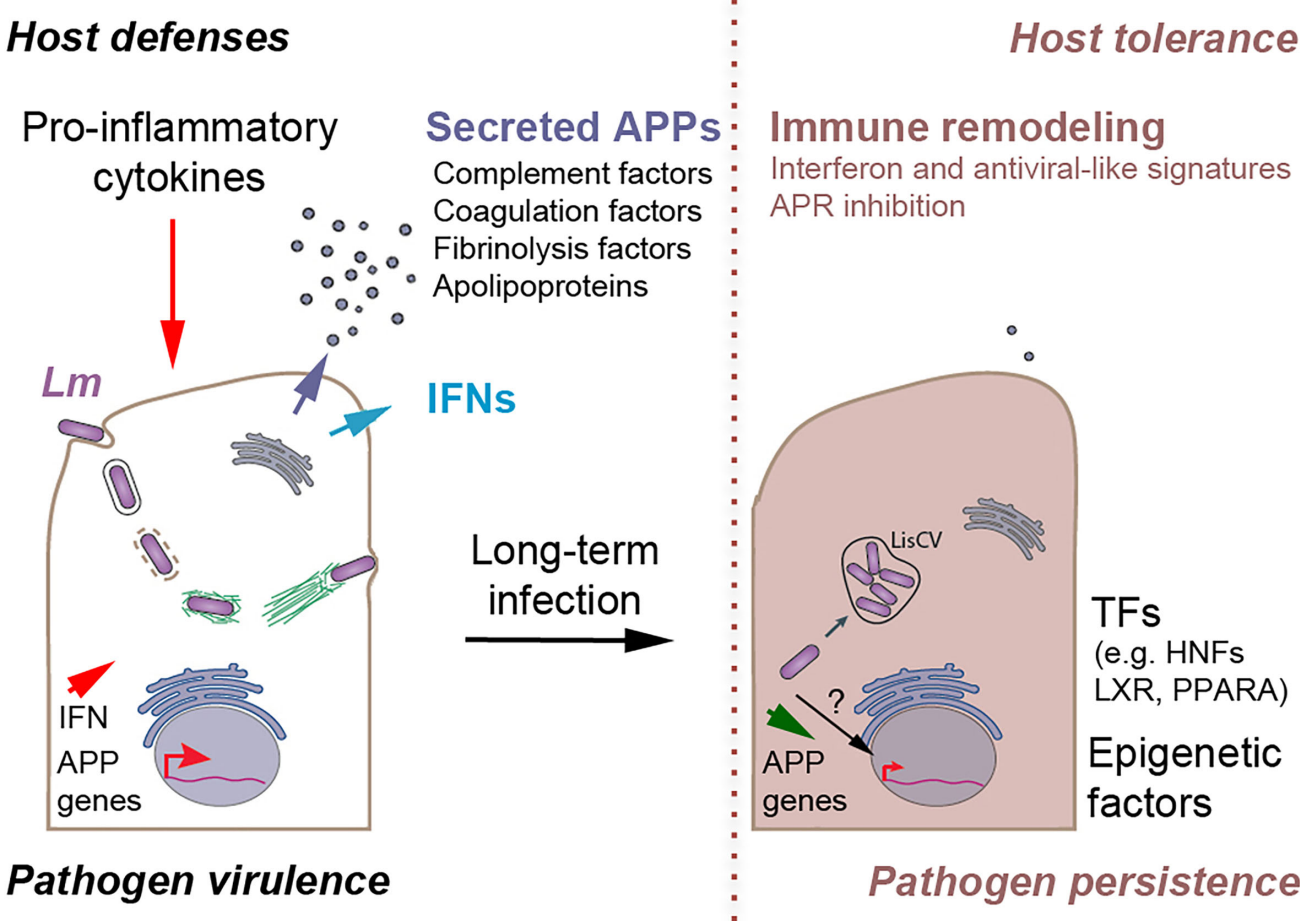
Proposed model of *Listeria-*mediated immune remodeling to promote persistent infection in hepatocytes. *Acute infection* (left). *L.* *monocytogenes (Lm)* invades hepatocytes (through the InlA/B-mediated entry pathways or through cell-to-cell spread from infected macrophages, not represented). During the active phases of the infection process, *Lm* produces virulence factors that enable bacteria to enter into the cytosol and spread from cell-to-cell by the ActA-mediated actin-motility process. Hepatocytes detect intracellular *Lm* and secrete type I/III interferons (IFNs) and other cytokines (not represented), stimulating immune cells. In turn, immune cells secrete pro-inflammatory cytokines, such as IL-6 and IL-1β, which activate the expression of genes encoding acute phase proteins (APP) in hepatocytes. Secreted APPs, which include complement, coagulation and fibrinolytic factors and a set of apolipoproteins, have antimicrobial and/or immunomodulatory effects that contribute to the clearance of *Listeria* infection. *Persistent infection* (right). Long-term *Lm* infection exacerbates interferon and anti-viral like responses and imposes a block on APP gene expression in hepatocytes, by an unknown mechanism possibly involving the inhibition of transcription factors (e.g. hepatocyte nuclear factors (HNFs), LXR, PPARA) and epigenetic regulators, leading to the decrease in APP secretion and inhibition of the APR. Concomitantly, bacteria stop expressing ActA and are engulfed in membrane compartments to form *Listeria*-containing vacuoles (LisCV), where they enter a persistent non-replicative phase. The deregulation of the hepatocyte-specific innate immune responses prevents the complete elimination of *Listeria* in the liver, favoring the creation of a long-term reservoir of intracellular persistent bacteria. In addition, it could modulate adaptive immunity to favor the tolerance of cells harboring dormant pathogens.

The ability of intracellular bacterial pathogens to hide long-term in host cells plays a key role in chronic or asymptomatic infections. However, the molecular mechanisms of this persistence are less well understood than those of virulence. Especially for *Listeria*, the concept of intracellular persistence is recent (Bierne et al., 2018) and its potential impact on host defenses remains unknown. Here, we established three cellular infection systems in human and murine hepatocytes to study how the host cell transcriptional landscape is remodeled once the bacteria enter a persistence stage in LisCVs. Convergent results indicate that this stage coincides with a profound deregulation of hepatic innate immune response genes, in particular a strong activation of the IFN response and repression of APR genes. We speculate that this immune deregulation may be propitious for asymptomatic *Listeria* carriage in the host (**Figure 8**).

### Hepatocyte Models to Study *Listeria* Persistent Infection

A number of studies have analyzed the response of mammalian cells infected with *Listeria* using transcriptomic approaches (**Supplementary Table S14**). Some have used cultured cells of myeloid, endothelial, or epithelial origin, but during a short time of infection, when the bacteria are in the active phase of replication and/or dissemination (i.e., 24 h or less). Others have used organ biopsies after infection in mice, thus revealing a response arising from different cell types. In particular, in transcriptomic analyses of *Listeria* infection in the mouse liver (**Supplementary Table S14**), the specific contribution of hepatocytes versus immune cells was not determined. In addition, while these studies have proven extremely valuable in defining *Listeria* virulence and host immune responses during acute infection, they did not reveal strategies that might allow this pathogen to maintain long-term infection without causing symptomatic disease. A major challenge in studying persistent bacteria is due to the fact that slow-growing bacteria could be rare within tissues and thus difficult to detect and study *in vivo.* It is therefore crucial to develop efficient *in vitro* culture systems to model persistent infection. We show here that HepG2, Huh7 and PMH are good models to achieve this goal, as bacteria can efficiently replicate, disseminate, and finally be captured inside LisCVs, without causing major cytotoxicity. This latter observation is consistent with *in vivo* studies showing that *Listeria* can replicate extensively in hepatocytes without causing major cell death, when neutrophil recruitment to the sites of infection in the liver is inhibited (Conlan and North, 1991; Appelberg and Leal, 2000). We exploited these *in vitro* models in order to identify, for the first time, a “meta-signature” associated with long-term *Listeria* infection in hepatocytes.

### The Intracellular Persistence Stage of *Listeria* in Hepatocytes Coincides With Inhibition of Complement and Coagulation Gene Expression

Hepatocytes play an important role in fighting bacterial infections, particularly as they are the primary source of many APPs, which they constitutively produce and overproduce in response to infection (Zhou et al., 2016). For example, complement C3 and fibrinogen (FBN), the central compounds of the complement and coagulation systems, are APPs constitutively and almost exclusively produced by hepatocytes, and their serum levels are significantly increased during the APR (Zhou et al., 2016). Hepatocytes produce other complement and coagulation proteins that, like C3 and FBN, contribute to pathogen clearance, activation of innate immune cells, and enhancement of the adaptive immune response (Amara et al., 2008; Merle et al., 2015; Reis et al., 2019). It is therefore not surprising that pathogenic bacteria have evolved various mechanisms to evade the complement and coagulation systems (Hovingh et al., 2016; Antoniak, 2018; Ermert et al., 2019). However, while known mechanisms primarily involve bacterial protein effectors (e.g., capsules, proteases, inhibitors), mechanisms involving transcriptional repression are essentially undocumented. There is a plethora of data showing that systemic bacterial infections induce APP gene expression, and this knowledge has long been exploited for diagnostic purposes, as serum APP levels can be used as prognostic markers (Markanday, 2015). In contrast, to our knowledge, data regarding the impact of persistent bacterial infections on APP gene expression are scarce. In a recent study involving *Mycobacterium tuberculosis* (Mtb), a paradigm for asymptomatic persistent infections, the blood plasma proteome of healthy subjects was compared to those with active tuberculosis (TB) or latent tuberculosis (LTBI). This revealed that the amount of several APPs decreased in the plasma of LTBI cases compared to TB cases or even healthy controls (Teklu et al., 2020), suggesting that in asymptomatic Mtb carriers the expression of some APPs is not induced or is reduced relative to basal levels, and that APP levels could be used to predict the risk of TB reactivation. Interestingly, decreased APP expression has been associated with recurrent bacterial infections. For example, C3 levels in women with recurrent urinary tract infections have been shown to be lower than in healthy women (Syukri et al., 2015). In addition, mutations that cause complement deficiencies predispose individuals to recurrent infections (e.g., by *Streptococcus pneumoniae, Neisseria meningitidis and Haemophilus influenza*e) (Ram et al., 2010). Single-nucleotide polymorphisms (SNPs) in complement genes have also been linked, through genome-wide association studies (GWAS), to disease susceptibility and outcome of bacterial infections (van den Broek et al., 2020).

Here we show that long-term *Listeria* infection in hepatocytes can have a profound impact on both the constitutive and cytokine stimulated expression of APP genes. Several studies have demonstrated the importance of the complement system (Calame et al., 2016) and the coagulation system (Davies et al., 1981; Mullarky et al., 2005; Nishanth et al., 2013) in protecting the host from *Listeria*. The transcriptional inhibition observed here could therefore be a previously unappreciated effect induced by the pathogen to enhance its long-term survival. Among targeted APPs, the decrease in the amount of C3 protein in the secretome of *Listeria*-infected hepatocytes, compared to non-infected hepatocytes, is particularly striking, given the critical role of C3 in the response to this bacterial infection (Calame et al., 2016). For instance, blocking the functional activity of C3 through the use of an anti-CR3 antibody inhibits neutrophil recruitment to *Listeria* infection foci and reduces liver tissue damage (Conlan and North, 1991; Appelberg and Leal, 2000). In addition, through activation of its receptors CR3 and CRIg, C3 contributes to phagocytosis and elimination of *Listeria* by macrophages (Calame et al., 2016). C3 also plays a role in adaptive immunity, being required for efficient T cell activation during murine listeriosis (Nakayama et al., 2009; Verschoor et al., 2011; Tan et al., 2014). Beyond its role on immune cells, C3 could also counteract bacterial infection within hepatocytes. Indeed, an intracellular activity of complement proteins [known as “complosome” (Arbore et al., 2017)] was recently identified and, interestingly, a study showed that coating *Listeria* with C3 induces autophagy in epithelial cells (Sorbara et al., 2018). Since C3 is abundantly produced by hepatocytes, it is possible that the intracellular C3 form targets *Listeria* within this cell type. Blocking C3 gene expression, while sheltering in the LisCV niche, could be a strategy employed by *Listeria* to avoid autophagy and persist in the hepatocyte. Thus, inhibition of C3 production could promote bacterial persistence by several means, such as preservation of the hepatocyte niche and limitation of intracellular bacterial destruction. With respect to other APPs, it is worth mentioning the impaired innate response to *Listeria* infection in *ApoE*-deficient mice, as another example of how a decrease in APP expression could impact the host response to this pathogen (Roselaar and Daugherty, 1998).

Inhibiting a network of APP genes, rather than individual APPs, is an effective strategy to concomitantly target multiple host defenses. To our knowledge, this has never been described before for an intracellular bacterium. *Listeria* infection represses the constitutive expression of APP genes in hepatocytes and, importantly, this repression is maintained under conditions of inflammation, after stimulation with the pro-inflammatory cytokines IL-6 and IL-1β. These results suggests that long-term *Listeria* infection could impose a transcriptional block, desensitizing APP gene expression from inflammatory stimuli. However, it is known that acute infection with *Listeria* in mice stimulates the production by immune cells of these pro-inflammatory cytokines, which in turn stimulate APP expression in hepatocytes (Kopf et al., 1994). Notably, one study showed that after intravenous inoculation of mice with a sublethal dose of *Listeria*, a homogeneous infection of mouse livers was observed on day 1, followed by a peak in bacterial loads on day 3 and a highly significant reduction on day 9 p.i., representing the expected process of bacterial clearance (Kummer et al., 2016). At the same time, IL-6 and IL-1β mRNA levels increased in the infected livers on day 1 and remained elevated on day 3, followed by a sharp decrease on day 9 p.i. Consecutively to the induction of pro-inflammatory cytokines, the amount of many APPs increased on day 1, and especially on day 3, and then dropped to an nonsignificant level on day 9 p.i. in infected livers compared with uninfected controls (Kummer et al., 2016). We propose a model that combines these results with our own (**Figure 8**). On the one hand, acute infection triggers a major defensive inflammatory response in the host, including expression of APPs, leading to the elimination of the majority of bacteria and infected hepatocytes. On the other hand, bacteria infecting hepatocytes control the expression, and thus the secretion, of APPs, which could counteract the massive activation of the APR and limit liver damage, while attenuating the immune response. We suggest that this process may prevent the complete elimination of *Listeria* in the liver, favoring the creation of a long-term reservoir of a small bacterial subpopulation that has entered a dormant state in LisCVs. Moreover, inhibition of APP gene expression could have profound implications for the development of effective adaptive immunity. Given the fact that the liver is a naturally immunotolerant organ (Zheng and Tian, 2019), downregulation of APPs could enhance host tolerance to *Listeria* persistent forms in asymptomatic carriers.

The mechanisms by which *Listeria* interferes with APP gene expression remain to be elucidated, but avenues of research exist based on the acquired knowledge of how this bacterium modulates host gene expression, e.g., by interfering with the signaling pathways that control TFs, impacting their stability or nuclear localization, or by acting on epigenetic regulators thus controlling the chromatin structure at target genes (Bierne and Hamon, 2020; Eldridge et al., 2020). Among possible hypotheses, *Listeria* could alter the functions of TFs essential for constitutive expression of APPs, such as hepatocyte nuclear factors (HNFs) HNF1α and HNF4α (Zhou et al., 2016). HNFs are particularly interesting candidates since they play a central role in the liver-specific transcriptional program. They coordinate the effect of various signaling pathways to fine-tune the expression of many liver genes (Odom et al., 2004), including those shown here to be downregulated by infection and involved in the complement and coagulation cascades or other facets of the APR (Zhou et al., 2016).

### The Intracellular Persistence Stage of *Listeria* in Human Hepatocytes Coincides With Inhibition of Cholesterol Metabolism Genes

HNFs regulate hepatic gene expression in concert with other ligand-dependent nuclear receptors such as LXR, FXR and PPARA that regulate lipid and bile acid metabolism (Shih et al., 2001; Odom et al., 2004; Wollam and Antebi, 2011; Yin et al., 2011). HNF4α and LXR, in turn, regulate the activity of SREBPs (Shimano and Sato, 2017), and are thus directly implicated in the regulation of cholesterol homeostasis. In Huh7 and HepG2 human hepatocytes, the pathway the most significantly inhibited by infection was the cholesterol biosynthesis gene network, with almost all genes of the pathway downregulated in both human hepatocyte models (**Figure 7**). The role of cholesterol metabolic reprogramming in persistent *Listeria* infection of epithelial cells deserves further investigation, given the role of cholesterol in the establishment of vacuolar niches for intracellular pathogens (Samanta et al., 2017) and of oxysterols in host immune functions (Cyster et al., 2014; Abrams et al., 2020). In addition, controlling cholesterol metabolism plays a key role in *Mtb* and *Helicobacter pylori* persistent infections (Pandey and Sassetti, 2008; Morey and Meyer, 2019). In PMH, long-term *Listeria* infection was not associated with the cholesterol biosynthesis pathway. Growth conditions, metabolic disparity between primary cell and carcinoma-derived cell lines and, importantly, species specificity, could account for the differences in the infection-associated transcriptional program between human hepatoma cell lines and PMH. However, it should be noted that some genes involved in cholesterol metabolism were also inhibited by infection in PMH, such as *Hmgcs2* and several target genes of LXR and FXR, including the cholesterol transporter gene *Abcg1*, the lipoprotein lipase gene *Lpl*, and several apolipoprotein genes, like in human hepatocytes.

### Interferon and Antiviral-Like Signatures of Long-Term *Listeria* Infection in the Hepatic Transcriptome

IFN-I and/or IFN-III expression is a hallmark of the epithelial cell response to viral or bacterial invasion, including by *Listeria* (Bierne et al., 2012; Dussurget et al., 2014). Here we show that human HepG2 hepatocytes respond to intracellular *Listeria* infection with significant IFN-III production, in agreement with our previous results (Bierne et al., 2012). PMH cells, on the other hand, respond with IFN-I expression. These differences are consistent with the species-specific expression of IFN-I and IFN-III genes in the liver, as well as of their specific receptors. Human hepatocytes express high levels of IFN-III and respond strongly to stimulation by this IFN, whereas murine hepatocytes do not respond to IFN-III and preferentially express IFN-I (Nakagawa et al., 2013; Hermant et al., 2014; Broggi et al., 2020). In addition, we report differential expression dynamics of IFN-I and IFN-III genes, which may have significance in the specific roles of the two classes of IFNs. This emphasizes the need to address the role of IFN-III in human listeriosis. As for Huh7 cells, they do not produce any IFN in response to *Listeria*, as previously observed when these cells are challenged with other IFN-triggering stimuli, such as dsRNA (Lanford et al., 2003), poly(I-C) (Li et al., 2005) or hepatitis C virus (Israelow et al., 2014). A possible explanation for this lack of response is that Huh7 cells have a defective TLR3 signaling pathway (Li et al., 2005). This deficiency can be useful in identifying IFN-independent processes. In particular, the downregulation of cholesterol biosynthesis gene expression in response to *Listeria* infection occurs in Huh7 in the absence of IFN. In contrast, IFN is involved in the downregulation of sterol biosynthesis during viral infections (Blanc et al., 2011).

Are IFN-I and IFN-III pathways beneficial or detrimental to persistent *Listeria* infection? The answer to this question is complex, since IFNs can have opposing effects on bacterial pathogens (Boxx and Cheng, 2016; Cohen and Parker, 2016; Ng et al., 2016; Alphonse et al., 2021). In fact, in mouse models of listeriosis, IFN-I has been implicated in both the restriction and promotion of infection (Dussurget et al., 2014; Boxx and Cheng, 2016), likely resulting from the pleiotropic roles of IFN-I in distinct cell environments and at different stages in the infectious process. Our data suggest that IFN responses do not result in an antibacterial effect in hepatocytes. Indeed, restoring a functional IFN response in Huh7 cells, by exogenous supply of IFN-β or IFN-λ1, does not produce a reduction in bacterial load at 3-day p.i. However, it is likely that IFN secretion by hepatocytes modulates infection *in vivo* by acting on immune cells. Interestingly, IFN-I promotes some chronic bacterial infections (Boxx and Cheng, 2016) and can lead to immunosuppression in chronic viral infections by inhibiting DC and T cell activation (Ng et al., 2016). In murine listeriosis, the IFN-I response has inhibitory effects on T cells (Carrero et al., 2004; Archer et al., 2014). The role of IFN-III in listeriosis has not yet been directly studied, but it should be noted that this IFN limits inflammation and leukocyte responses that are detrimental to epithelial barrier integrity (Broggi et al., 2020). Based on this, we propose the hypothesis that excessive IFN signaling could promote cellular conditions that support bacterial persistence by contributing to immune suppression and tissue tolerance (**Figure 8**).

Proteins encoded by the genes activated downstream of IFN (ISGs) are an important family of innate immunity factors produced in response to microbial infections (Schneider et al., 2014), including listeriosis (Dussurget et al., 2014). Like APPs, ISGs have various functions and are activated in a network, allowing for the simultaneous induction of diverse responses. The striking result of our study is that long-term infection with *Listeria* in human (HepG2) and murine (PMH) hepatocytes leads to a prominent ISG signature, astonishingly mimetic of an antiviral response. In addition, we identified 16 ISGs whose expression is enhanced in all hepatocyte models at 3 days p.i. (**Table 1**). Since *Listeria* infection does not induce IFN expression in Huh7 cells, these 16 ISGs are, in this context, activated by alternative pathways to IFN signaling. Indeed, *CCL5, CXCL10*, *PMAIP1*, *IFIT2, RSAD2* and *ISG15* can be activated independently of IFNs (Varley et al., 2003; Werts et al., 2007; Knowlton et al., 2012; Ashley et al., 2019). In particular, *ISG15* expression is known to be induced independently of IFN-I/III signaling upon *Listeria* infection and its product, the ubiquitin-like modifier ISG15, inhibits *Listeria* infection in fibroblasts (Radoshevich et al., 2015). The role of ISG15 in hepatocytes has not yet been directly addressed, but the study of a mouse model of increased ISGylation suggests an important role of ISG15 in the liver, including metabolic processes and autophagy (Zhang et al., 2019). Interestingly, hyper-ISGylation is associated with larger infection foci in the liver, suggesting that uncontrolled ISGylation could promote *Listeria* survival in this organ.

### Concluding Remarks

The inhibition of the APR and exacerbation of interferon responses in hepatocytes sheds new light on the mechanisms by which *Listeria* might escape the host immune system to create a niche of persistence in the liver. Our results open up new questions about the cell biology of *Listeria* infection, for which the cellular models presented here would prove useful. For example, what is the role of intracellular activities of complement proteins, ISG products, and cholesterol metabolism, on the fate of cytosolic or vacuolar bacteria? What are the transcriptional and/or epigenetic mechanisms behind the deregulation of the identified genes? This study also raises the question of the role of APPs, IFN-I and IFN-III *in vivo*, especially in the physiology of the asymptomatic phase of *Listeria* infections. However, no relevant animal model to study a long-lasting silent *Listeria* infection is yet available. Exploiting the results of our study might guide the development of such models, through genetic modifications or the use of drugs targeting host genes involved in the APR and IFN signaling. In addition, as SNPs and other mutations in complement and IFN genes have been linked to immune deregulation and susceptibility to infection, GWAS in humans or animals with listeriosis would be valuable in the investigation of the potential roles of APR and IFN pathway-related genes in the susceptibility to *Listeria* infections.

## Data Availability Statement

The RNA-seq data presented in the study are deposited in the Gene Expression Omnibus (GEO) repository (https://www.ncbi.nlm.nih.gov/geo/), accession numbers GSE184697, GSE184729 and GSE184808. The MS proteomics data have been deposited in the ProteomeXchange Consortium via the PRIDE partner repository (https://www.ebi.ac.uk/pride/), accession number PXD027154.

## Ethics Statement

Mice were housed under specific pathogen free conditions in the local animal facility of IERP (INRAE, Jouy-en-Josas) in agreement with animal welfare guidelines. The animal house was maintained on a 12-hour light/dark cycle. The animal study was reviewed and approved by the local ethics committee, the COMETHEA (“Comité d’Ethique en Expérimentation Animale du Centre INRAE de Jouy en Josas et AgroParisTech”), under the registration numbers 19-08 and by the French Ministry of Higher Education and Research (APAFIS #20380-2019060315249683 v1) and were performed in accordance with European directive 2010/63/EU.

## Author Contributions

Investigation and methodology: ND, AP, CH, KG, CA, AD-B, and HB. Formal analysis and data curation: ND, LJ, KG, CH, and HB. Visualization: ND and HB. Resources: LG, PS, and HB. Funding acquisition: HB, LG, and CA. Writing - original draft preparation: HB. Writing - review and editing: ND, AP, CA, PS, LG, and HB. Supervision: HB, AP, and CA. Conceptualization and administration: HB. All authors contributed to the article and approved the submitted version.

## Funding

This work was funded by grants from FFRC FCRF (grant 2017) to HB and LG, ANR PERMALI (N°ANR-20-CE35-0001-01), ANR TheraEpi (N°ANR-20-PAMR-0011) and iXcore Foundation (2015) to HB, and INRAE-MICA division (AAP 2019) to CA.

## Conflict of Interest

The authors declare that the research was conducted in the absence of any commercial or financial relationships that could be construed as a potential conflict of interest.

## Publisher’s Note

All claims expressed in this article are solely those of the authors and do not necessarily represent those of their affiliated organizations, or those of the publisher, the editors and the reviewers. Any product that may be evaluated in this article, or claim that may be made by its manufacturer, is not guaranteed or endorsed by the publisher.
