# Corrigendum: An Immunomodulatory Transcriptional Signature Associated With Persistent *Listeria* Infection in Hepatocytes

**DOI:** 10.3389/fcimb.2022.911320

**Published:** 2022-06-14

**Authors:** Natalie Descoeudres, Luc Jouneau, Céline Henry, Kevin Gorrichon, Aurélie Derré-Bobillot, Pascale Serror, Laura Lee Gillespie, Cristel Archambaud, Alessandro Pagliuso, Hélène Bierne

**Affiliations:** ^1^ Université Paris-Saclay, INRAE, AgroParisTech, Micalis Institute, Jouy-en-Josas, France; ^2^ Université Paris-Saclay, INRAE, Virologie et Immunologie Moléculaires, Jouy-en-Josas, France; ^3^ Université Paris-Saclay, Institut de Biologie Intégrative de la Cellule, CEA, CNRS UMR 9198, Université Paris-Sud, Gif-sur-Yvette, France; ^4^ Terry Fox Cancer Research Laboratories, Division of BioMedical Sciences, Faculty of Medicine, Memorial University of Newfoundland, St. John’s, NL, Canada

**Keywords:** *Listeria monocytogenes*, liver, acute phase response, interferon, persistence, innate immunity, cholesterol, transcriptomics

In the original article, there was a mistake in **Supplementary Figure S1**, as published. **Supplementary Figure S1B**was mistakenly replaced by **Figure S2**, which appears thus duplicated, and the word “Hoetschst” was mispelled, the correct spelling of this word being “Hoechst”. The corrected **Supplementary Figure S1B** is shown below.

In addition, there was a mistake in **Figure 1** and **Supplementary S2**, as published. The word “Hoetscht” was misspelled. The correct spelling of this word is “Hoechst”. The corrected **Figure 1** and **Supplementary S2** are shown below.

The authors apologize for these errors and state that this does not change the scientific conclusions of the article in any way. The original article has been updated.

**Figure 1:**
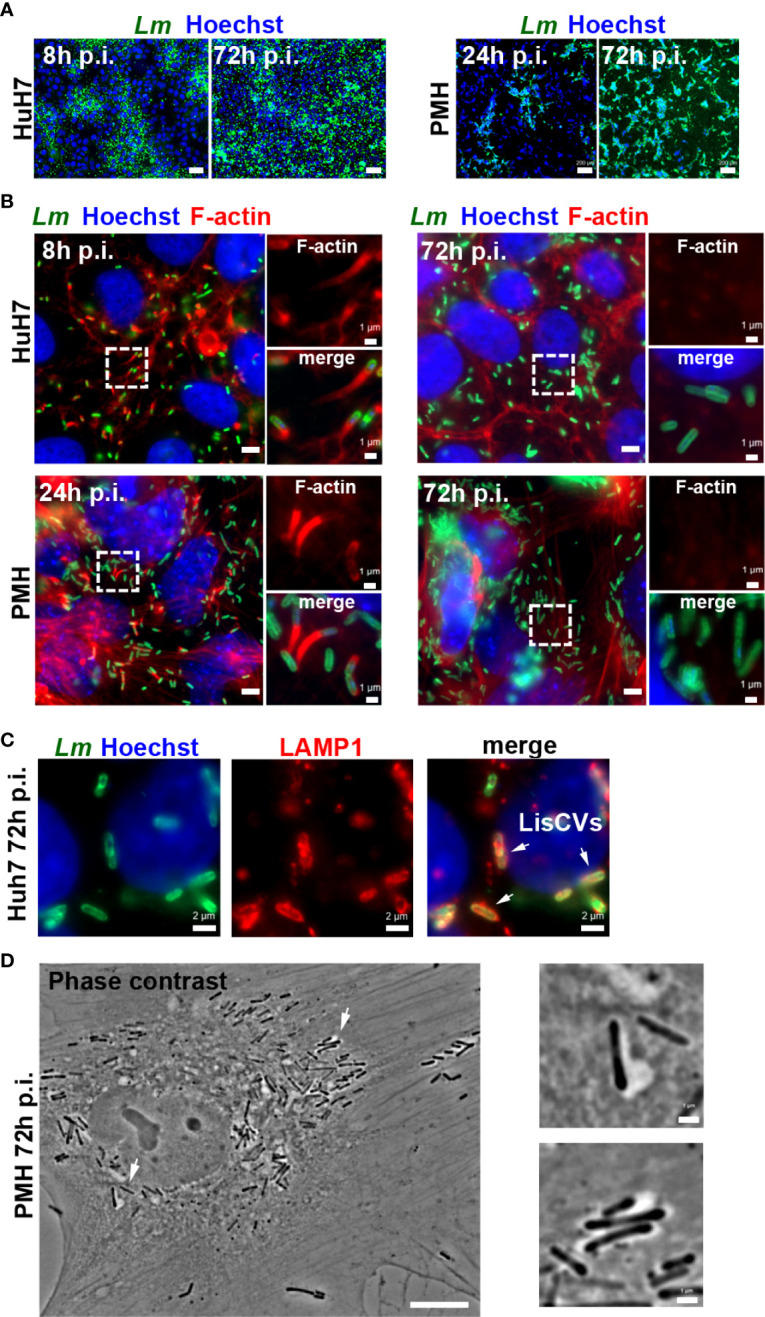
Optimization of hepatocyte culture systems for modeling persistent *Listeria* infection. Different cell seeding conditions, MOI and *Listeria* strains (EGDe or 10403S) were tested to obtain optimal long-term *Listeria* infection of HepG2 (see **Supplementary Figure S1**), Huh7 or PMH. Infected cells were examined at day 1 (d1) and at day 3 (d3) by immunofluorescence microscopy: representative examples under optimized conditions are shown. **(A)** Low magnification micrographs of Huh7 cells infected with EGDe strain (MOI=1-5) or PMH infected with 10403S strain (MOI=10) for the indicated time. Images are overlays of *Listeria* (green) and Hoechst (blue) signals (bars: 50 μm, Huh7, or 200 μm, PMH). **(B)** High magnification micrographs of infected Huh7 or PMH showing *Listeria* (green), F-actin (red) and Hoechst (blue) signals. Bars: 5 μm. Boxed regions enlarged on the right show F-actin (top) or merged signals (bottom), highlighting actin-positive bacteria at d1 and actin-negative bacteria at d3 (bars: 1 μm). **(C)** Micrographs of an infected Huh7 cell at d3, showing *Listeria* (green), LAMP1 (red) and Hoechst (blue) signals. Arrows indicate 3 examples of LisCVs. **(D)** Phase contrast image of an infected PMH at d3 (bars: 10 μm). Arrows indicate 2 examples of bacteria within vacuoles, shown at a higher magnification on the right (bars: 1 μm).

## Publisher’s Note

All claims expressed in this article are solely those of the authors and do not necessarily represent those of their affiliated organizations, or those of the publisher, the editors and the reviewers. Any product that may be evaluated in this article, or claim that may be made by its manufacturer, is not guaranteed or endorsed by the publisher.

