# Supplementary material for: An Immunomodulatory Transcriptional Signature Associated With Persistent Listeria Infection in Hepatocytes
Source: Front Cell Infect Microbiol. 2021 Nov 10;11:761945. doi: 10.3389/fcimb.2021.761945 (PMC8631403; doi:10.3389/fcimb.2021.761945)
Supplement: Supplementary file 1 [file DataSheet_1.pdf]

Supplementary Material

1 Supplementary Materials and Methods

1.1 Transcriptome analysis

1.1.1 RNA-sequencing

Directional RNA-seq libraries of infected and non-infected cell RNA were assembled using 500-1300 ng total RNA (as determined by Qubit assay (Invitrogen)) using the TruSeq® mRNA Stranded Library Prep kit (Illumina) which includes polyA-selection. The RNA-seq libraries were monitored for quality on the Bioanalyzer 2100 using an Agilent High Sensitivity DNA Kit. Libraries were pooled in equimolar proportions and sequenced in paired-end 50-35 bp runs on an Illumina NextSeq500 instrument, using NextSeq 500 High Output 75 cycles kits. Demultiplexing was performed (bcl2fastq2 V2.2.18.12) and adapters were trimmed with Cutadapt (v1.15); only reads longer than 10 bp were kept. TopHat (version 2.1.1) was used for alignment on the reference genomes: Ensembl-98 human genome (HepG2 and Huh7 samples) or Ensembl-100 GRCm38 mouse genome (PMH samples). Data were evaluated through principal component analysis and hierarchical clustering after transformation of the count data using RLOG function. Dendograms were built using Euclidian distance function and Ward criterion as linkage function. Normalization and differential analysis were carried out using the DESeq2 package, with a pre-filter step to remove genes with low counts (sum of all replicates counts<10, in all conditions compared). Results were considered statistically significant with adjusted $p<0.05$, with correction according to the Benjamini and Hochberg's procedure.

1.1.2 Functional gene analysis

Gene pathways analysis was performed on differentially expressed protein-coding genes using DAVID (Huang da et al. 2009) version 6.8 (http://david.abcc.ncifcrf.gov), KEGG (Kanehisa et al. 2000) (http://www.genome.jp/kegg/pathway.html) and QIAGEN's Ingenuity Pathway Analysis (IPA®, QIAGEN Redwood City). A p value <0.05 was considered to indicate a statistically significant difference. Analysis using the interferome database (Rusinova et al. 2013) was performed by selecting "hepatocytes" as the cell system, "all species", and a fold change value greater or equal to 2.

1.1.3 RT-qPCR validation

Each RNA-seq analysis was validated by RT-qPCR with *YWHAZ*, *PPIA*, or *Pdk1* used to normalize gene expression in infected relative to non-infected HepG2, HuH7 and PMH, respectively. Pearson correlation analysis was applied to the log2 FC of 14 significantly deregulated genes obtained by RNA-seq and RT-qPCR. Pearson's correlation coefficients (R^2) of 0.9365, 0.9582, and 0.9728 were obtained for HepG2, Huh7 and PMH respectively, indicating very strong correlation and thus the precision of both methods in the relative quantification of gene expression.

1.1.4 Data Availability

The RNA-seq data presented in the study are deposited in the Gene Expression Omnibus (GEO) repository (https://www.ncbi.nlm.nih.gov/geo/), accession numbers GSE184697 (*Lm* EGDe versus non-infected HepG2, 72 h), GSE184729 (*Lm* EGDe versus non-infected Huh7, 72 h) and GSE184808 (*Lm* 10403S versus non-infected PMH, 72 h).

1.2 Proteome analysis

1.2.1 Sample preparation

For the proteome analysis, 5 µg of protein extract were used for a short migration 1D gel electrophoresis (NuPAGE® 4-12 % Bis-Tris Gel, Novex). Proteins were visualized using Coomassie G-250 (SimplyBlue™ SafeStain, Invitrogen) and the whole-colored part of each lane was cut into small pieces. The gel pieces were destained using Solvent A (10 % v/v acetic acid, 40 % v/v ethanol) and Solvent B (50 % v/v 50 mM ammonium bicarbonate, 50 % v/v acetonitrile). The proteins contained in the gel were reduced by 10 mM dithiothreitol (Sigma) and alkylated by 55 mM iodoacetamide (Sigma). The proteins were digested with 200 ng of trypsin (Promega) and afterwards extracted using a solution of 0.5 % v/v trifluoroacetic acid and 50 % v/v acetonitrile. The peptides were dried completely using a concentrator (Savant™ SPD121D, Thermo Fisher Scientific) and taken up in 50 µl loading buffer (0.08 % v/v trifluoroacetic acid, 2 % v/v acetonitrile) for LC-MS/MS proteome analysis (4 µl = 400 ng peptides per injection).

1.2.2 Liquid Chromatography – Mass Spectrometry

Mass spectrometry was performed on the PAPPSO platform (MICALIS, INRAE, Jouy-en-Josas, France; http://pappso.inrae.fr). We used an Orbitrap Fusion^{TM} Lumos^{TM} Tribrid^{TM} (Thermo Fisher Scientific) coupled to an UltiMate™ 3000 RSLCnano System (Thermo Fisher Scientific). A 4 µl sample was loaded at 20 µl/min on a precolumn (µ-Precolumn, 300 µm i.d x 5 mm, C18 PepMap100, 5 µm, 100 Å, Thermo Fisher) and washed with loading buffer. After 3 min, the precolumn cartridge was connected to the separating column (Acclaim PepMap®, 75 µm x 500 mm, C18, 3 µm, 100 Å, Thermo Fisher). Buffer A consisted of 0.1 % formic acid in 2 % acetonitrile and buffer B of 0.1 % formic acid in 80 % acetonitrile. The peptide separation analysis was achieved at 300 nl/min with a linear gradient from 0 to 30 % buffer B for 50 min and 30 % to 40 % for 5 min. One run took 66 min, including the regeneration step at 98 % buffer B. Positive ionization (1.6 kV ionization potential) and capillary transfer (270 °C) were performed with a liquid junction and a capillary probe (SilicaTip™ Emitter, 10 µm, New Objective). Peptide ions were analyzed using Data Dependent Acquisition (DDA) with HCD (Higher-energy Collisional Dissociation) mode and the machine settings were as follows: 1) full MS scan in Orbitrap (scan range [m/z] = 400–1600), and 2) MS/MS using HCD (30 % collision energy) in Orbitrap (AGC target = 5.0×10^4, max. injection time = 150 ms, data type = centroid). Analyzed charge states were set to 2-4, the dynamic exclusion to 100 s and the intensity threshold was fixed at 2.0×10^4.

1.2.3 Data Analyses

Identification. The *Homo sapiens* database (Uniprot, version 2021 taxonomy identifier = 9606, 20 396 entries) was searched by using X!TandemPipeline version 0.4.24 (Langella et al., 2017). The proteome identification was run with a precursor and a fragment mass tolerance of 10 ppm. Enzymatic cleavage rules were set to trypsin digestion ("after Arg and Lys, unless Pro follows directly after") and no semi-enzymatic cleavage rules were allowed. The fix modification was set to cysteine carbamidomethylation and methionine oxidation was considered as a potential modification. In a second pass, N-terminal acetylation was added as another potential modification, whereas all other previous settings were retained. The identified proteins were filtered as follows: 1) peptide E-value < 0.05 with a minimum of 2 peptides per protein and 2) a protein E-value of $< 10^{-4}$.

Quantification. Proteins were quantified by the spectral counting (SC) method. MassChroqR (version 0.5.2), an R package developed by PAPPSO platform (http://pappso.inrae.fr) was used to check the quality of data and practice statistical analysis in proteomic. The abundance in number of spectra was

modeled using the following generalized mixed model (GLM) with a Poisson distribution, as already described (Millan-Oropeza et al., 2017). Filters consist in removing proteins showing low numbers of spectra (Filter was at least 3 spectra per protein, for example, if cutoff = 3, all the proteins quantified with only 0, 1, 2 spectra in each of the injections will be removed) and the selection criteria is the ratio between the minimal and the maximal mean abundance values computed for a factor or a combination of factors of interest (here, cutoff =1.5). Protein abundance change were detected by analysis of variance (ANOVA) using a Chi-square test. The obtained p-values were adjusted for multiple testing by the Benjamini-Hochberg approach. Adjusted p-values obtained from ANOVA for the proteome was considered significant below a value of 0.01. For HepG2 samples, 486 proteins (sub groups) were detected and quantified. 214 proteins remained after filter application, and statistical analysis (adj p-value < 0.01) selected 42 proteins as less present in infected samples compared to uninfected samples. For Huh7 cells, 502 proteins (sub groups) were detected and quantified. 337 proteins remained after filter application and statistical analysis (adj p-value < 0.01), selected 153 proteins as less present in infected samples compared to uninfected samples. The data were then crossed with the transcriptome data to sort proteins whose corresponding gene was significantly downregulated in infected cells compared to uninfected cells.

1.2.4 Data Deposition

The MS proteomics data have been deposited to the ProteomeXchange Consortium via the PRIDE (Perez-Riverol et al. 2019) repository: **https://www.ebi.ac.uk/pride/archive/projects/PXD027154**

1.3 References

Huang da W, Sherman BT, Lempicki RA. Systematic and integrative analysis of large gene lists using DAVID bioinformatics resources. *Nat Protoc* (2009) 4(1):44-57. PubMed PMID: 19131956.

Kanehisa M, Goto S. KEGG: kyoto encyclopedia of genes and genomes. *Nucleic Acids Res* (2000) 28(1):27-30. Epub 1999/12/11. doi: 10.1093/nar/28.1.27. PubMed PMID: 10592173.

Langella O, Valot B, Balliau T, Blein-Nicolas M, Bonhomme L, Zivy M. X!TandemPipeline: A Tool to Manage Sequence Redundancy for Protein Inference and Phosphosite Identification. *J. Proteome Res.* (2017). 2 (16) 494-503. PubMed PMID: 27990826

Millan-Oropeza A, Henry C, Blein-Nicolas M, Aubert-Frambourg A, Moussa F, Bleton J, Virolle MJ. Quantitative Proteomics Analysis Confirmed Oxidative Metabolism Predominates in Streptomyces coelicolor versus Glycolytic Metabolism in Streptomyces lividans. *J Proteome Res* (2017). 7;16(7):2597-2613. PubMed PMID: 28560880

Perez-Riverol Y, Csordas A, Bai J, Bernal-Llinares M, Hewapathirana S, Kundu DJ, Inuganti A, Griss J, Mayer G, Eisenacher M, Pérez E, Uszkoreit J, Pfeuffer J, Sachsenberg T, Yilmaz S, Tiwary S, Cox J, Audain E, Walzer M, Jarnuczak AF, Ternent T, Brazma A, Vizcaíno JA (2019). The PRIDE database and related tools and resources in 2019: improving support for quantification data. *Nucleic Acids Res* 47(D1):D442-D450. PubMed PMID: 30395289.

Rusinova I, Forster S, Yu S, Kannan A, Masse M, Cumming H, et al. Interferome v2.0: an updated database of annotated interferon-regulated genes. *Nucleic Acids Res* (2013) 41(Database issue):D1040-6. Epub 2012/12/04.. PubMed PMID: 23203888

Vizcaíno J.A, Csordas A, del-Toro N, Dianes JA, Griss J, Lavidas I, Mayer G, Perez-Riverol Y, Reisinger F, Ternent T, Xu QW, Wang R, Hermjakob H. 2016 update of the PRIDE database and its related tools. *Nucleic Acids Research* (2016) 4;44(D1):D447-56. PubMed PMID: 2652772

2 Supplementary Figures and Tables

2.1 Supplementary Figures

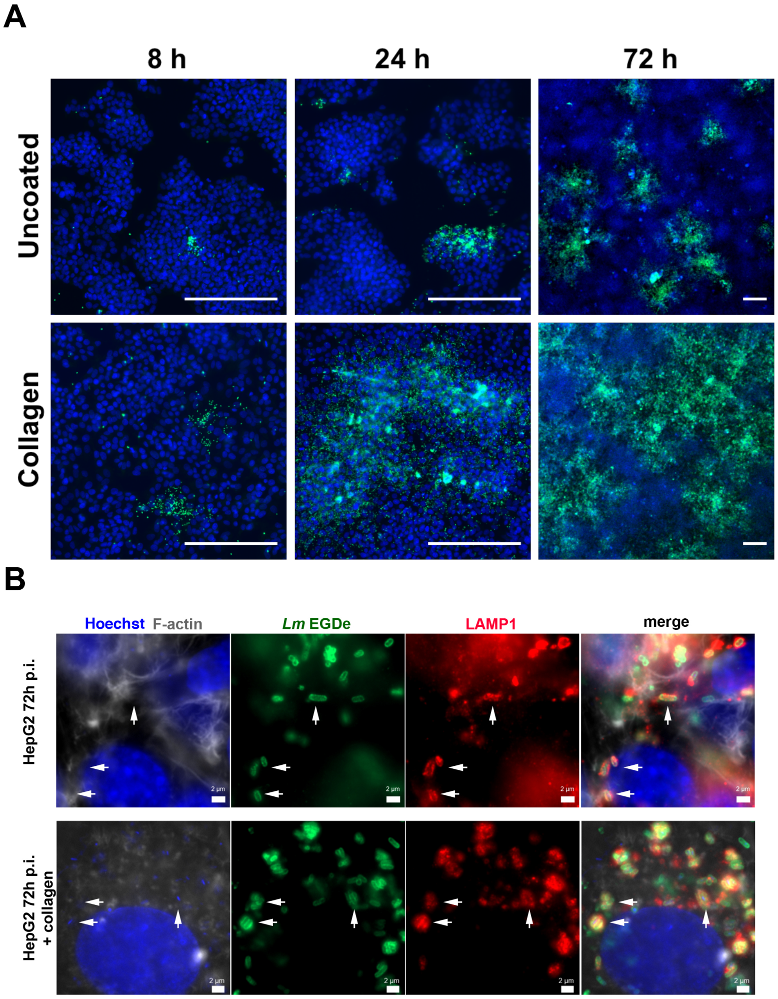

Supplementary Figure 1. Collagen coating increases bacterial spreading during 3-day *Listeria* infection in HepG2 cells. (A) HepG2 cells grown on uncoated or collagen-coated coverslips were infected with *Listeria* strain EGDe (MOI~1-5) and examined by immunofluorescence microscopy. **(A)** Low-magnification micrographs showing infected HepG2 cells at 8 h, 24 h and 72 h p.i., stained with Hoechst (to label nuclei, in blue) and *Listeria* antibodies (to label bacteria, in green). Monolayer growth of HepG2 on collagen decreases isolated clusters of cells, resulting in higher bacterial intercellular spreading at 8 h and 24 h p.i. and an increased number of infected cells at 72 h p.i. (bars: 200 µm). **(B)** High magnification micrographs showing cells at 72 h p.i. stained with Hoechst to label nuclei (blue), AF-647-phalloidin to label F-actin (white), *Listeria* antibody to label bacteria (green) and LAMP1 antibody to label LisCVs (red). Bacteria in LisCVs, which are LAMP1-positive, are observed both in HepG2 grown on uncoated (top) or collagen-coated coverslips (bottom).

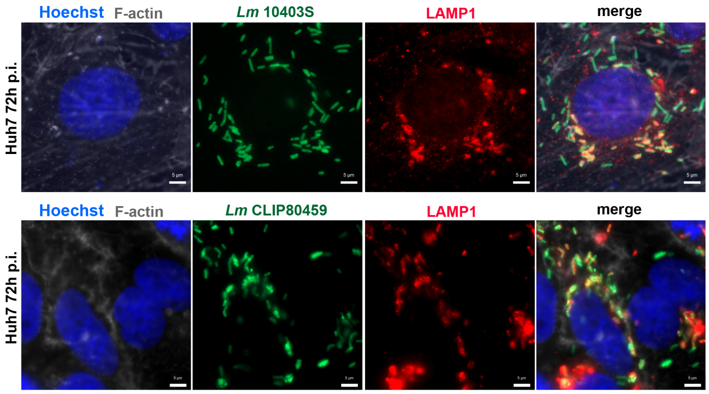

Supplementary Figure 2. Formation of LisCVs in Huh7 cells is strain-independent. Huh7 cells were infected with laboratory strain 10403S or clinical strain CLIP80459 for 72 h and examined by immunofluorescence microscopy. High magnification micrographs show cells stained with Hoechst to label nuclei (blue), AF-647-phalloidin to label F-actin (white), *Listeria* antibody to label bacteria (green) and LAMP1 antibody to label LisCVs (red). LAMP1-positive bacteria are observed with both strains. Bars: 5 µm.

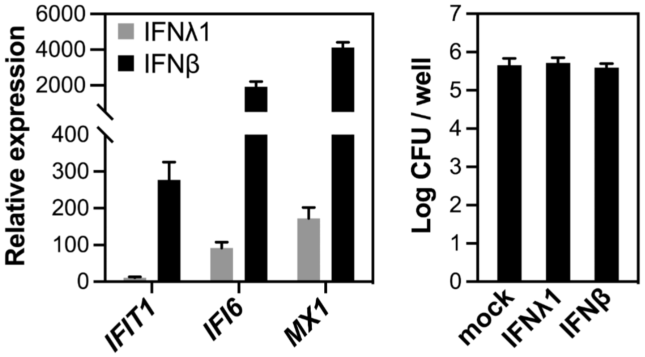

Supplementary Figure 3. Activation of IFN responses with recombinant IFN-β or IFN-λ1 does not change bacterial loads in Huh7 cells. Huh7 cells were infected with *Listeria* EGDe (MOI~1-5) for 24 h to let bacteria carry out the early phases of infection, and subsequently treated with either recombinant IFN-β (3 ng/ml; ~800 IU /ml) or IFN-λ1 (100 ng/ml) for an additional 48 h. IFN treatment activated the expression of ISGs as evaluated by RT-qPCR analysis of the transcript levels of *IFIT1, IFI6* and *MX1*, relative to mock-stimulated cells and normalized to *YWHAZ* (**A**), but did not result in the reduction of the number of intracellular bacteria at 72 h p.i., compared to untreated cells **(B).** For all experiments, histograms represent means and standard deviations of triplicate experiments.

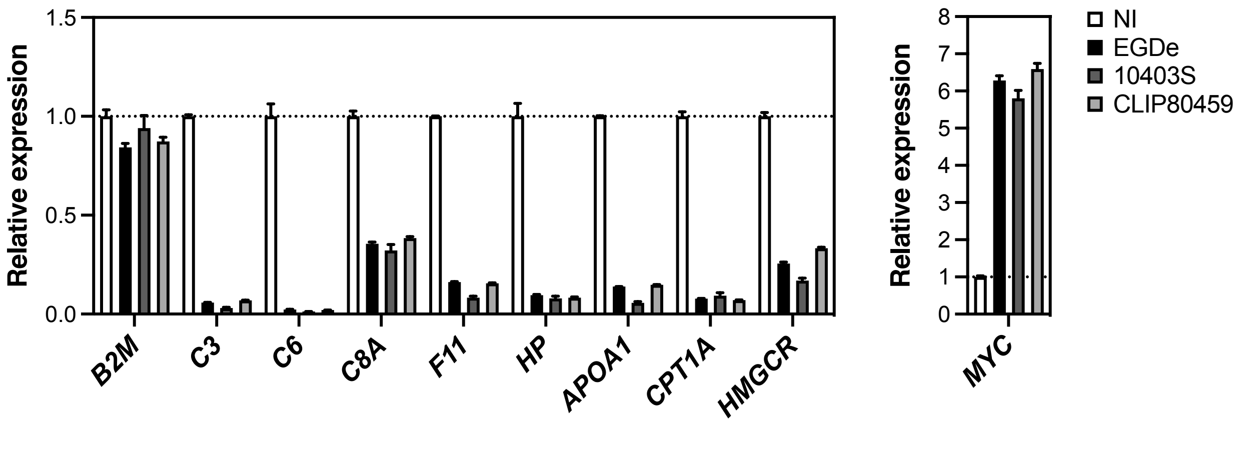

Supplementary Figure 4. Dysregulation of APP and lipid-metabolism gene expression in hepatocytes is strain-independent. RT-qPCR analysis of transcript levels of representative APP genes (C3, C6, C8A, F11, HP), or lipid metabolism-associated genes (*APOA1*, *CPT1A*, *HMGCR)*, in Huh7 cells infected with *Listeria* EGDe, 10403S, or CLIP80459 at 72h p.i., relative to non-infected cells (NI) cells. *PPIA* expression was used to normalize target gene expression and a control gene (*B2M*) whose expression was unaffected by infection, and an upregulated gene (*MYC*) are included. Histograms represent means and standard deviations of triplicate experiments.

2.2 Supplementary Tables

All supplementary Tables are attached as a single Supplementary Excel file, and are as follows:

Table S1. List of RT-qPCR primers.

Table S2. Protein-coding genes upregulated in HepG2 cells infected with *L. monocytogenes* EGDe for 72h, relative to non-infected HepG2 cells.

Table S3. Protein-coding genes downregulated in HepG2 cells infected with *L. monocytogenes* EGDe for 72h, relative to non-infected HepG2 cells.

Table S4. Protein-coding genes upregulated in Huh7 cells infected with *L. monocytogenes* EGDe for 72h, relative to non-infected Huh7 cells.

Table S5. Protein-coding genes downregulated in Huh7 cells infected with *L. monocytogenes* EGDe for 72h, relative to non-infected Huh7 cells.

Table S6. Protein-coding genes upregulated in PMH infected with *L. monocytogenes* 10403S for 72h, relative to non-infected PMH**.**

Table S7. Protein-coding genes downregulated in PMH infected with *L. monocytogenes* 10403S for 72h, relative to non-infected PMH.

Table S8. Genes upregulated in both HepG2 cells and PMH infected with *L. monocytogenes* for 72h.

Table S9. Gene Ontology of Biological Processes (GO-BP) and Kyoto Encyclopedia of Genes and Genomes (KEGG) pathway enrichment analysis of genes upregulated in HepG2, Huh7 or PMH infected with *L. monocytogenes* for 72h.

Table S10. Kyoto Encyclopedia of Genes and Genomes (KEGG) pathway enrichment analysis of genes downregulated in HepG2, Huh7 or PMH infected with *L. monocytogenes* for 72h.

Table S11. Genes downregulated by 72h *L. monocytogenes* infection in all HepG2, Huh7 and PMH datasets (A) or intersecting Huh7 and HepG2 datasets (B), or Huh7 and PMH datasets (C).

Table S12. Proteins identified as significantly less abundant in the conditioned medium of 72h infected HepG2 (A) or Huh7 (B) cells, compared to non-infected cells.

Table S13. Ingenuity Upstream Regulator Analysis (URA) on downregulated genes common to both human Huh7 and murine PMH models (A) or both human Huh7 and HepG2 models (B).

Table S14. List of references providing information on host transcriptional responses to *L. monocytogenes* infection in cellular models *in vitro* (A) or in mouse models *in vivo* (B).
