# Supplementary figures and images for: Corrigendum: An Immunomodulatory Transcriptional Signature Associated With Persistent Listeria Infection in Hepatocytes
Source: Front Cell Infect Microbiol. 2022 Jun 14;12:911320. doi: 10.3389/fcimb.2022.911320 (PMC9237639; doi:10.3389/fcimb.2022.911320)

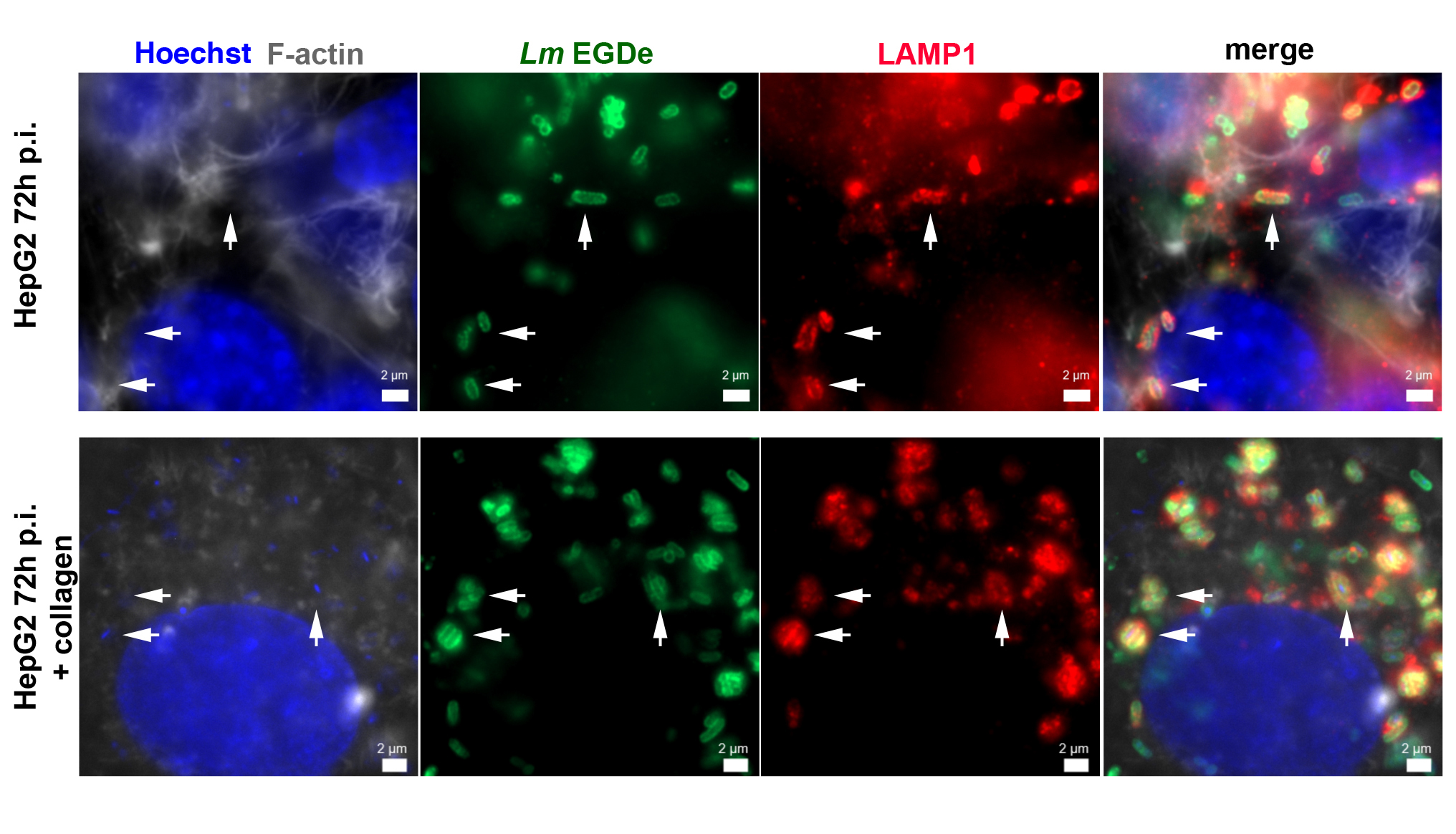

Supplement: Supplementary file 1 [file Image_1.jpg]

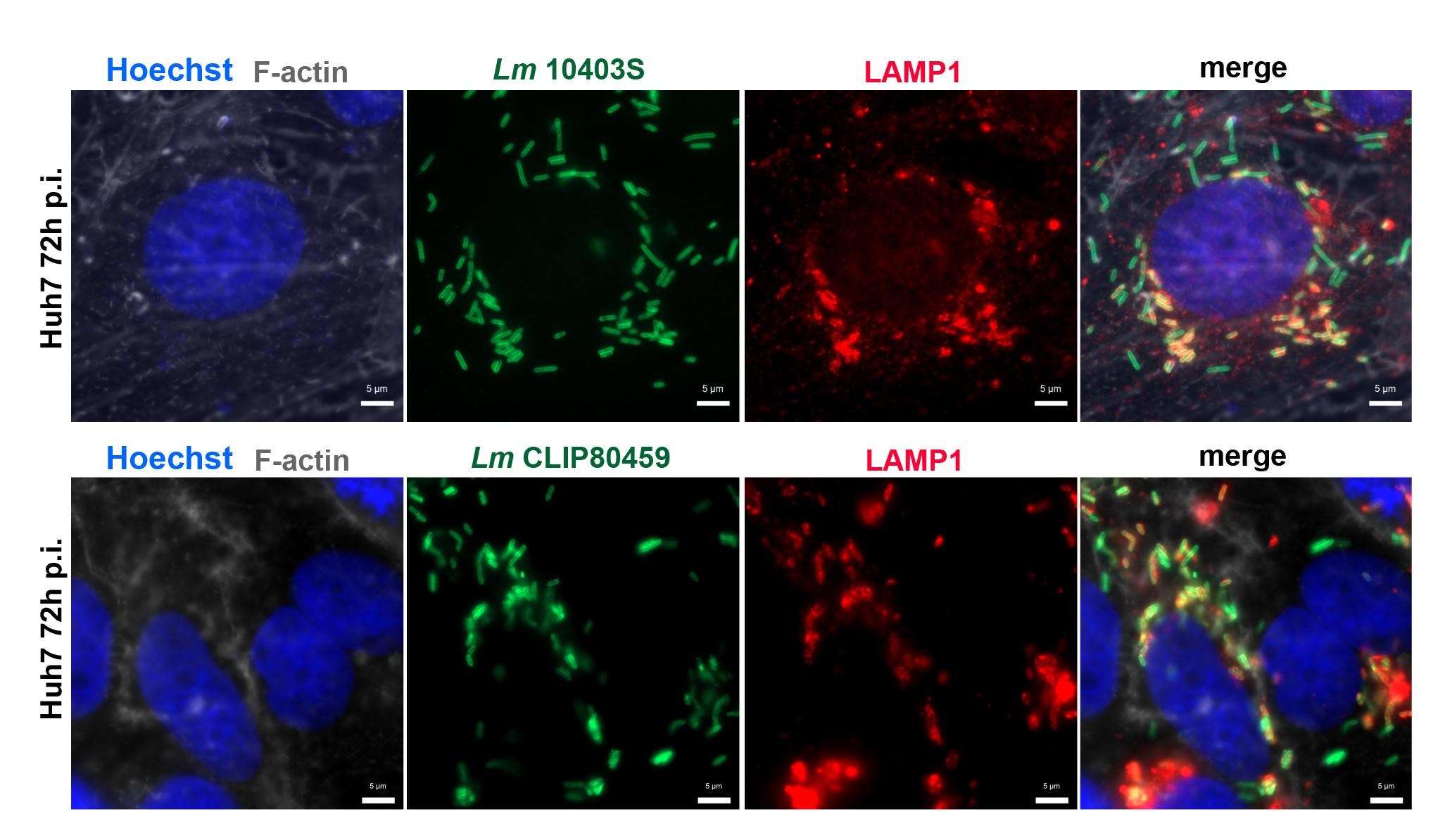

Supplement: Supplementary file 2 [file Image_2.jpg]
